# Systematic analysis of IGF2BP family members in non-small-cell lung cancer

**DOI:** 10.1186/s40246-024-00632-6

**Published:** 2024-06-12

**Authors:** Liping Gong, Qin Liu, Ming Jia, Xifeng Sun

**Affiliations:** 1 Department of Academic Research, The Secondary Hospital, Cheeloo College of Medicine, Shandong University, Jinan, 250033 China; 2 Department of Cancer Center, The Secondary Hospital, Cheeloo College of Medicine, Shandong University, Jinan, 250033 China; 3 Institute of Medical Sciences, The Secondary Hospital, Cheeloo College of Medicine, Shandong University, 247 Beiyuan Street, Jinan, Shandong 250033 P.R. China

**Keywords:** IGF2BP, NSCLC, Survival, Cancer immunity, Meta-analysis

## Abstract

**Background:**

The insulin-like growth factor-2 mRNA-binding proteins 1, 2, and 3 (IGF2BP1, IGF2BP2, and IGF2BP3) are known to be involved in tumorigenesis, metastasis, prognosis, and cancer immunity in various human cancers, including non-small cell lung cancer (NSCLC). However, the literature on NSCLC largely omits the specific context of lung squamous cell carcinoma (LUSC), an oversight we aim to address.

**Methods:**

Our study evaluated the differential expression of IGF2BP family members in tumors and normal tissues. Meta-analyses were conducted to assess the prognostic value of IGF2BPs in lung adenocarcinoma (LUAD) and LUSC. Additionally, correlations between IGF2BPs and tumor immune cell infiltration, mutation characteristics, chemotherapy sensitivity, and tumor mutation burden (TMB) were investigated. GSEA was utilized to delineate biological processes and pathways associated with IGF2BPs.

**Results:**

IGF2BP2 and IGF2BP3 expression were found to be upregulated in LUSC patients. IGF2BP2 mRNA levels were correlated with cancer immunity in both LUSC and LUAD patients. A higher frequency of gene mutations was observed in different IGF2BP1/2/3 expression groups in LUAD compared to LUSC. Meta-analyses revealed a significant negative correlation between overall survival (OS) and IGF2BP2/3 expression in LUAD patients but not in LUSC patients. GSEA indicated a positive association between VEGF and IGF2BP family genes in LUAD, while matrix metallopeptidase activity was inversely correlated with IGF2BP family genes in LUSC. Several chemotherapy drugs showed significantly lower IC50 values in high IGF2BP expression groups in both LUAD and LUSC.

**Conclusion:**

Our findings indicated that IGF2BPs play different roles in LUAD and LUSC. This divergence highlights the need for tailored therapeutic strategies and prognostic tools, cognizant of the unique molecular profiles of LUAD and LUSC.

**Supplementary Information:**

The online version contains supplementary material available at 10.1186/s40246-024-00632-6.

## Introduction

In 2020, lung cancer emerged as the second most frequently diagnosed cancer and maintained its status as the primary cause of mortality attributed to cancer [1]. It is categorized into two histological subtypes: small-cell lung cancer (SCLC) and non-small-cell lung cancer (NSCLC). NSCLC, which comprises about 85% of all lung cancer cases, is primarily classified into lung squamous cell carcinoma (LUSC) and lung adenocarcinoma (LUAD) [2, 3]. Significant research efforts have focused on unreaveling the mechanisms underlying the development, progression, and metastasis of NSCLC in an attempt to decrease its mortality rate [4, 5]. Over the last decade, advancements in immune and targeted therapies have significantly altered the treatment paradigm for NSCLC [6–9]. Despite these developments, the effectiveness of targeted therapy is limited, as not all patients with driver gene mutations benefit from such treatments. Additionally, only a minority of NSCLC patients exhibit favorable responses and enhanced long-term survival following immunotherapy. Consequently, the overall cure and survival rates for NSCLC, especially in metastatic stages, remain suboptimal. Identifying new, highly specific, and sensitive biomarkers, along with novel molecular targets, is crucial not only for elucidating the molecular mechanisms of NSCLC but also for improving treatment outcomes.

Insulin-like growth factor 2 mRNA-binding proteins (IGF2BPs) are part of an evolutionarily preserved family of single-stranded RNA-binding oncofetal proteins. This family includes IGF2BP1, IGF2BP2, and IGF2BP3 [10, 11]. These proteins have been demonstrated to function as m6A readers, stabilizing target mRNAs such as S1PR3 [12], c-MYC [13], PEG10 [14], SOX2 [15], LYPD1 [16], YES1 [17], MGAT5 [18], and SRF [19], leading to various biological effects. IGF2BPs have also been implicated in tumorigenesis across various cancers and their active involvement in cell functions in tumor-derived cells, including cell polarization, adhesion, and migration [11]. IGF2BPs have been found to promote an aggressive phenotype in tumor-derived cells, enhancing tumor growth and drug resistance [20]. Previous research has established a correlation between IGF2BP3 and the promotion of bladder cancer cell proliferation through the activation of the JAK/STAT signaling pathway [21]. Similarly, IGF2BP1 is known to enhance invasive growth driven by SRC/MAPK in ovarian cancer cells [22]. In pancreatic cancer, upregulation of IGF2BP2 promotes cell proliferation via the PI3K/Akt signaling pathway [23]. In the context of NSCLC, several studies have underscored the importance of IGF2BPs in oncogenesis and cancer development [10]. Researchers have shown that the ALKBH5-IGF2BPs axis promotes cell proliferation and tumorigenicity, leading to an unfavorable prognosis in NSCLC [24]. Research has reported that circNDUFB2 facilitates the interaction between TRIM25 and IGF2BPs, resulting in the ubiquitination and degradation of IGF2BPs, thereby inhibiting the growth and metastasis of NSCLC [10]. Additionally, IGF2BPs have been found to be upregulated in LUAD patients, correlating with poor overall survival. Furthermore, Hao et al. indicated that IGF2BPs mitigate the detrimental effects of irradiation on LUAD by upregulating VANGL1 [25].

Recent research has revealed that IGF2BPs may exert a substantial influence on the immune response within the tumor microenvironment. Elcheva et al. have demonstrated that reducing the expression of IGF2BPs enhances the expression of interferon beta-stimulated genes and increases the infiltration levels of NK cells and tumor-associated myeloid cells in melanoma mouse models [26]. Specifically, IGF2BP2 promotes the polarization of tumor-associated macrophages (TAMs) towards the M2 phenotype [27], while IGF2BP3 promotes the polarization of TAMs to an immunosuppressive phenotype. Moreover, a peptide epitope derived from IGF2BP3 has been identified to stimulate CD8 + T cells, generating a potent and specific immune response against cancer cells [28, 29]. IGF2BP3 has also been reported to stabilize PD-L1 mRNA expression, thereby inhibiting the effects of cytotoxic T cells [30]and suppressing CD8 + T cell infiltration in NSCLC [31]. It also appears to diminish NK cell-mediated cytotoxicity via facilitating the decay of the stress-induced ligand ULBP2 mRNA [32]. In addition, a pan-cancer analysis has revealed significant associations between IGF2BP family expression profiles and microsatellite instability (MSI), infiltration of certain immune cells, tumor mutational burden (TMB), and various immune checkpoint biomarkers [33]. However, most studies focusing on NSCLC have not included LUSC. Given the substantial differences in biological behavior and treatment strategies between LUAD and LUSC, the specific mechanisms of IGF2BP family members in LUSC remain unclear. Our study aimed to analyze and compare the expression and mutations of different IGF2BPs and their associations with prognostic value, the immunomicroenvironment, and drug sensitivity in both LUAD and LUSC patients. This approach may unveil the molecular mechanisms that contribute to the NSCLC tumorigenesis and identify new prognostic and therapeutic targets.

## Materials and methods

### Clinical sample

This study utilized a total of 30 samples, which included 10 paraffin-embedded LUAD samples, 10 paraffin-embedded LUSC samples, and 10 paraffin-embedded normal lung tissue samples. These samples were collected from the Department of Cancer Center at the Second Hospital of Shandong University in July 2023. The Institutional Research Ethics Committee of Shandong University granted ethical approval and obtained each patient’s consent prior to the research.

### Quantitative real-time PCR (qRT-PCR)

Total RNA from the paraffin-embedded tissue samples was extracted using the RNAprep Pure FFPE kit (TIANGEN, Beijing, China). First-strand DNA synthesis was performed using Omniscript Reverse Transcriptase (TIANGEN, Beijing, China), adhering to the manufacturer’s instructions. The primers used were as follows: IGF2BP1 forward primer 5’- TGAAGCTGGAGACCCACATA-3’, reverse primer 5’-GGGTCTGGTCTCTTGGTACT-3’; IGF2BP2 forward primer 5’-GTTGGTGCCATCATCGGAAAGG − 3’, reverse primer 5’-TGGATGGTGACAGGCTTCTCTG-3’; IGF2BP3 forward primer 5’-GCTCTATCAGTCGGTGCCATCATC-3’, reverse primer 5’-GCCTTGAACTGAGCCTCTGGTG-3’; beta-actin forward primer 5’- CTCCATCCTGGCCTCGCTGT-3’, reverse primer 5’-GCTGTCACCTTCACCGTTCC-3’. All reactions were conducted in accordance with the guidelines provided by the manufacturer. These involved the use of UltraSYBR Mixture (including ROX; Beijing CoWin Bioscience Co., Ltd.), 250 nM primer (Invitrogen), and 100 ng of cDNA in a 20 µl reaction volume. Each individual sample was analyzed in quadruplicate across three independent tests. The results were standardized using beta-actin, an endogenous internal control.

### Histologic and immunohistochemical analysis

Pathological sections from LUAD (10 sets), LUSC (10 sets), and non-cancer tissue (10 sets) were obtained from the Second Hospital of Shandong University. The paraffin-embedded tissues were dissected into 4 μm thickness slices, then deparaffinized with a gradient xylene solution and rehydrated with a gradient ethanol solution. Following this, the slices were subjected to a 10-minute treatment at 37 °C with 3% hydrogen peroxide to suppress the activity of endogenous peroxidase. Non-specific binding sites were blocked using 10% bovine serum albumin at room temperature for an hour. Overnight incubation at 4 °C followed, using rabbit polyclonal antibodies against IGF2BP1, 2, and 3 (Catalog numbers: 22803-1-AP/11601-1-AP/14642-1-AP, Proteintech, Wuhan, China) at a 1/200 dilution. This was followed by a 2-hour incubation with HRP-conjugated secondary antibody at room temperature. The slices were then stained with 3-diaminobenzidine and counterstained with hematoxylin, with cytoplasmic staining being evaluated. Two independent pathologists observed and photographed the sections using a microscope.

### Data collection

The clinical characteristics and gene expression profiles (HTSeq-FPKM) for LUAD and LUSC patients were obtained from the Cancer Genome Atlas (TCGA) database through the GDC hub of the UCSC Xena website (http://xena.ucsc.edu/public, accessed on July 15, 2023). In cases where a single patient had multiple samples in the dataset, the tumor sample from the primary lesion was selected. The normalized gene expression values conversion to transcripts per million (TPM) and underwent logarithmic transformation (log2 (TPM + 1)). The gene symbols were mapped to the ensemble IDs utilizing the “org.Hs.eg.db” and “clusterProfiler” R packages. The divergent expression of IGF2BP1/2/3 between tumor and normal tissues across LUAD and LUSC was analyzed using TCGA datasets.

cBioPortal is a platform for analyzing multidimensional cancer genomics. It houses over 200 cancer genomics studies from TCGA [34]. In our study, we examined the genomic profiles of the IGF2BP family members, including structural variants and copy-number alterations. Kaplan–Meier plots were utilized to illustrate the genetic variants within the IGF2BP family and their association with overall survival (OS) and disease-free survival (DFS) in patients diagnosed with LUAD and LUSC. To determine the significance of differences between the survival curves, the log-rank test was utilized.

The mutation annotation format (maf) file of the simple nucleotide variation data (workflow type: VarScan2 Variant Aggregation and Masking) for the TCGA_LUAD and TCGA_LUSC cohorts was obtained from the Genomic Data Commons (GDC) database (https://portal.gdc.cancer.gov/). The data was processed using the “maftools” package in R to calculate the total mutation burden of each NSCLC sample.

### Correlation between IGF2BP family genes and tumor immunity

TIMER (https://cistrome.shinyapps.io/timer/) was utilized to assess the mRNA expression and mutation levels of IGF2BP family genes in LUAD and LUSC, as well as their associations with immune infiltrating cells (B cells, CD4 + and CD8 + T cells, neutrophils, macrophages, and dendritic cells). The correlations between the expression of IGF2BP family genes and key genes targeted in immunotherapy were also evaluated. The expression levels of these genes were quantified as log2 TPM. We utilized the “ESTIMATE” package in R to compute the immunological score, stromal score, and ESTIMATE score to evaluate the tumor microenvironment of each individual patient [35]. Throughout these calculations, all parameters in the R equation were set to their default values.

### Meta-analyses

The survival analysis of IGF2BPs was evaluated using the OSluca program (https://bioinfo.henu.edu.cn/LUCA/LUCAList.jsp) [36], which included 35 expression datasets from 5741 lung cancer patients. Patients were divided into groups (IGF2BPs high vs. IGF2BPs low) by the median mRNA levels of IGF2BPs. Meta-analyses and sensitivity analyses were performed using the “meta” package in R. Hazard ratios and confidence intervals were calculated.

### Gene set enrichment analysis (GSEA)

The biological functions of IGF2BP1/2/3 in LUAD and LUSC were investigated using GSEA. NSCLC samples were divided into groups (IGF2BPs high vs. IGF2BPs low) using the median mRNA levels of IGF2BPs as cutoff values to undergo the analysis. This analysis was performed utilizing the “clusterProfiler” package in R, based on the Kyoto Encyclopedia of Genes and Genomes (KEGG) and Gene Ontology (GO). Only the top five GO functions and KEGG pathways with the smallest *P* values were presented.

### Statistical analysis

GraphPad Prism (version 9.5.1, GraphPad Software, Inc., San Diego, CA, USA) and R (version 4.0.5, R Foundation for Statistical Computing, Vienna, Austria) were utilized to perform all statistical analyses. To evaluate the association between IGF2BPs, levels of immune cell infiltration, and critical immune target genes, Spearman correlation analysis was utilized. Only two factors with the absolute value of correlation coefficient over than 0.2 was considered as relevant factors. To compare the numerical values of two groups, the Wilcoxon test was utilized. For multiple comparison adjustments involving data points that were frequently utilized in hypothesis testing, the Bonferroni correction was implemented and we used *P*_adjust_ to address the results. When multiple comparison adjustment was not needed, *P* < 0.05 was considered statistically significant. In other cases, only *P*_adjust_ < 0.05 was considered statistically significant. The data analysis process of the entire study is shown in Supplemental Fig. 1.

## Results

### IGF2BP gene expression is elevated in patients with NSCLC

**Fig. 1:**
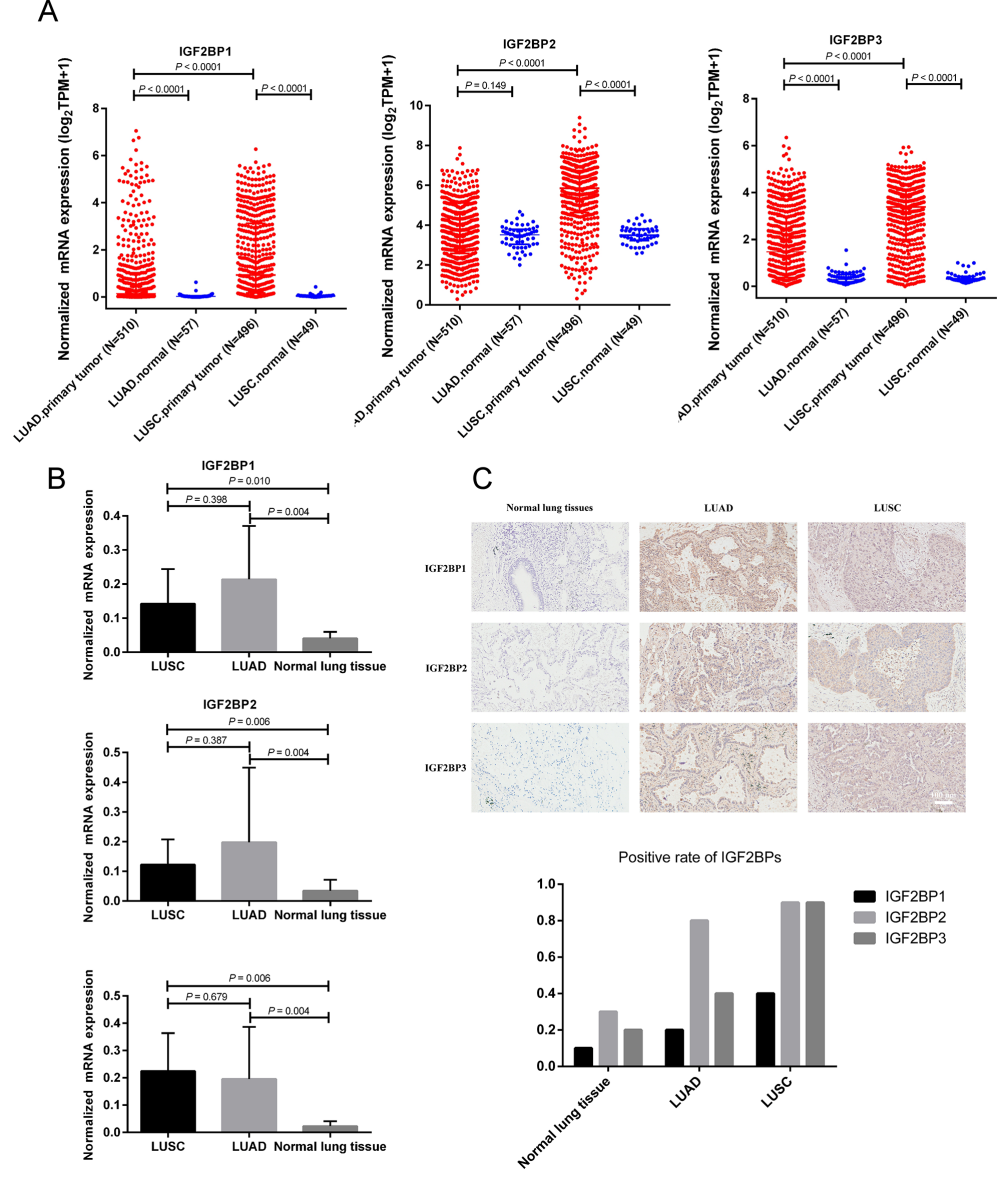
The expression of diverse IGF2BP family members in tumor and normal tissues. (**A**) The mRNA expression levels of IGF2BP family genes in tumor tissues and adjacent normal tissues from TCGA datasets. (**B**) QRT-PCR results histograms showed that the expression of IGF2BPs mRNA in LUAD, LUSC and normal tissues in collected samples. (**C**) The protein expression of IGF2BPs in collected samples

The IGF2BP family genes are located at specific genomic sites [37]. They encode three proteins (IGF2BP1/2/3) that are similar in the order and spacing of their domains. These proteins feature two RNA-recognition motifs in their N-terminal regions and four hnRNP-K homology domains in the C-terminal regions [11]. Utilizing the TCGA database, we analyzed transcriptome-seq data for the IGF2BP family in NSCLC and corresponding normal tissues. The mRNA expression levels of IGF2BP1/2/3 were significantly upregulated in LUSC tissues. In contrast, in LUAD, only the mRNA expressions of IGF2BP1 and IGF2BP3 were significantly elevated in tumor tissues compared to adjacent normal tissues. Figure 1A illustrated these differences in mRNA expression for the IGF2BP family members across LUAD, LUSC tissues and normal lung tissues. Specifically, in the LUAD group, IGF2BP1 (*P*_adjust_ < 0.0001) and IGF2BP3 (*P*_adjust_ < 0.0001) were both significantly upregulated compared to the normal group, while expression of IGF2BP2 was not found to be statistical difference (*P*_adjust_ = 0.447). In the LUSC group, all three genes (IGF2BP1, IGF2BP2, and IGF2BP3) were significantly upregulated compared to the normal group (*P*_adjust_ < 0.0001 for each). Besides, IGF2BPs mRNA levels in LUSC were higher than those in LUAD (*P*_adjust_ < 0.0001 for each). This analysis included 510 primary LUAD and 497 primary LUSC samples, compared with 57 and 49 adjacent lung tissues, respectively.

Then, we utilized qRT-PCR and immunohistochemical staining to evaluate the mRNA and protein expression levels of IGF2BPs in LUAD, LUSC, and 10 non-cancer lung tissues. The mRNA expressions of IGF2BP1/2/3 in LUAD were significantly higher than those in normal lung tissues (*P*_adjust_ = 0.012, 0.012, and 0.012, respectively) (Fig. 1B). In LUSC, IGF2BP1/2/3 mRNA levels were also significantly higher compared to normal lung tissues (*P*_adjust_ = 0.030, 0.018, and 0.018, respectively). The mRNA expression levels of IGF2BP1/2/3 between LUAD and LUSC were statistically similar (*P*_adjust_ > 0.999 for each). Protein expression of IGF2BPs displayed heterogeneity across LUAD, LUSC, and normal lung tissues (Fig. 1C). The expression levels of IGF2BP2 and IGF2BP3 proteins were significantly higher in LUSC compared to normal lung tissues (*P*_adjust_ = 0.019 and 0.005, respectively) (Table 1). In LUAD, the expression rate of IGF2BP2 protein was not found to be statistically different to normal tissues (*P*_adjust_ = 0.072). The expression rates of IGF2BP1 protein in both LUAD and LUSC appeared higher than in normal tissues, yet this difference was not statistically significant, potentially due to the limited sample size.

**Table 1:** IGF2BPs expression by IHC in NSCLC and normal lung tissue

Type	Num	IGF2BP1		*P* ^*a*^	*P* ^*b*^	IGF2BP2		*P* ^*a*^	*P* ^*b*^	IGF2BP3		*P* ^*a*^	*P* ^*b*^
		-	+	0.271		-	+	**0.009**		-	+	**0.006**	
LUAD	10	8	2		LUAD vs. NT 0.999	2	8		LUAD vs. NT 0.072	6	4		LUAD vs. NT 0.927
LUSC	10	6	4		LUSC vs. NT 0.363	1	9		**LUSC vs. NT 0.019**	1	9		**LUSC vs. NT 0.005**
NT	10	9	1		LUAD vs. LUSC 0.987	7	3		LUAD vs. LUSC 0.999	8	2		LUAD vs. LUSC 0.057

^a^:*P* value for chi-square test

^b^:Adjusted *P* value for partitions of chi-square test

Abbreviations: LUAD: lung adenocarcinoma, LUSC: lung squamous cell carcinoma, NT: normal lung tissue. The results were in **bold**, if *P* < 0.05

### Association between IGF2BP family and tumor immune system in NSCLC

**Fig. 2:**
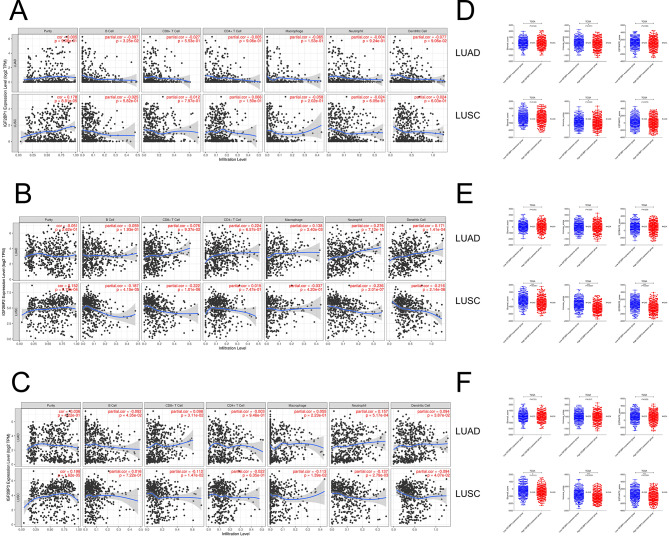
Correlation of IGF2BP1/2/3 expression with immune infiltration levels and immune scores in LUAD and LUSC. Correlation analysis of infiltrating levels of immune cells (CD8 + T cell, CD4 + T cell, B cell, macrophage, neutrophil and dendritic cell) and IGF2BP1 (**A**), IGF2BP2 (**B**) IGF2BP3 (**C**) mRNA expression levels in LUAD and LUSC. Association between ESTIMATE immune scores and IGF2BP1 (**D**), IGF2BP2 (**E**) IGF2BP3 (**F**) mRNA expression levels in LUAD and LUSC from TCGA dataset.*P < 0.05; **P < 0.01; ***P < 0.001

We further explored the influence of IGF2BP family genes on the immune system in NSCLC. We discovered distinct correlations in LUAD and LUSC. Specifically, IGF2BP2 mRNA levels demonstrated a weakly positive correlation with the infiltration of CD4 + T cells (Cor = 0.224, *P*_adjust_ < 0.0001), and neutrophils (Cor = 0.276, *P*_adjust_ < 0.0001) in LUAD. Conversely, in LUSC, IGF2BP2 mRNA levels were weakly negatively correlated with CD8 + T cells (Cor=-0.222, *P*_adjust_ < 0.0001), neutrophils (Cor=-0.236, *P*_adjust_ < 0.0001), and dendritic cells (Cor=-0.216, *P*_adjust_ < 0.0001), as illustrated in Fig. 2B. However, no significant associations were observed between the mRNA expression levels of IGF2BP1 and IGF2BP3 and various immune cells in NSCLC (Fig. 2A, C). Additionally, changes in immune cell infiltration levels in LUSC were found to be associated with the copy number variations of IGF2BP2. High amplification of the IGF2BP2 gene was inversely related to the infiltration of B cells, CD8 + T cells, CD4 + T cells, macrophages, neutrophils, and dendritic cells (*P*_adjust_ = 0.047, 0.001, 0.017, 0.010, < 0.0001, and < 0.0001, respectively) (Supplemental Fig. 2B). This trend was not evident in LUAD. For IGF2BP1 and IGF2BP3, no significant correlation was found between their copy numbers and immune cell infiltration levels in either LUAD or LUSC (Supplemental Fig. 2A, C). Furthermore, immune scores, which reflect the immune microenvironment, were analyzed in relation to IGF2BP family genes. In LUSC, immune scores including Stromal score, Immune score and ESTIMATE score were lower in the high-expression groups of IGF2BP2 (*P*_adjust_ < 0.0001 for each) (Fig. 2E) and IGF2BP3 (*P*_adjust_ = 0.030, < 0.0001, and < 0.0001, respectively) (Fig. 2F) compared to the low-expression groups. However, no significant differences in immune scores were observed between these groups in LUAD (Fig. 2D, E, F).

We further investigated the relationship between the mRNA expression of IGF2BP family genes and key immunotherapy targets, focusing on their potential roles in immunotherapy response efficacy. In LUAD, we observed IGF2BP2 expression showed a weakly positive correlation with several immunotherapy targets, including PD-1 (Cor = 0.227, *P*_adjust_ < 0.0001), PD-L1 (Cor = 0.303, *P*_adjust_ < 0.0001), LAG-3 (Cor = 0.234, *P*_adjust_ < 0.0001), and TIGIT (Cor = 0.225, *P*_adjust_ < 0.0001) (Supplemental Fig. 3A-C). In contrast, IGF2BP2 expression was only weakly negatively associated with the expression of TIM-3 (Cor=-0.215 *P*_adjust_ < 0.0001) in LUSC (Supplemental Fig. 3D-F). These findings indicated varied associations between IGF2BP family gene expression and immunotherapy targets in LUAD and LUSC, suggesting potential implications for immunotherapy responses in these cancer subtypes.

### Correlation between IGF2BP family and tumor mutation burden

**Fig. 3:**
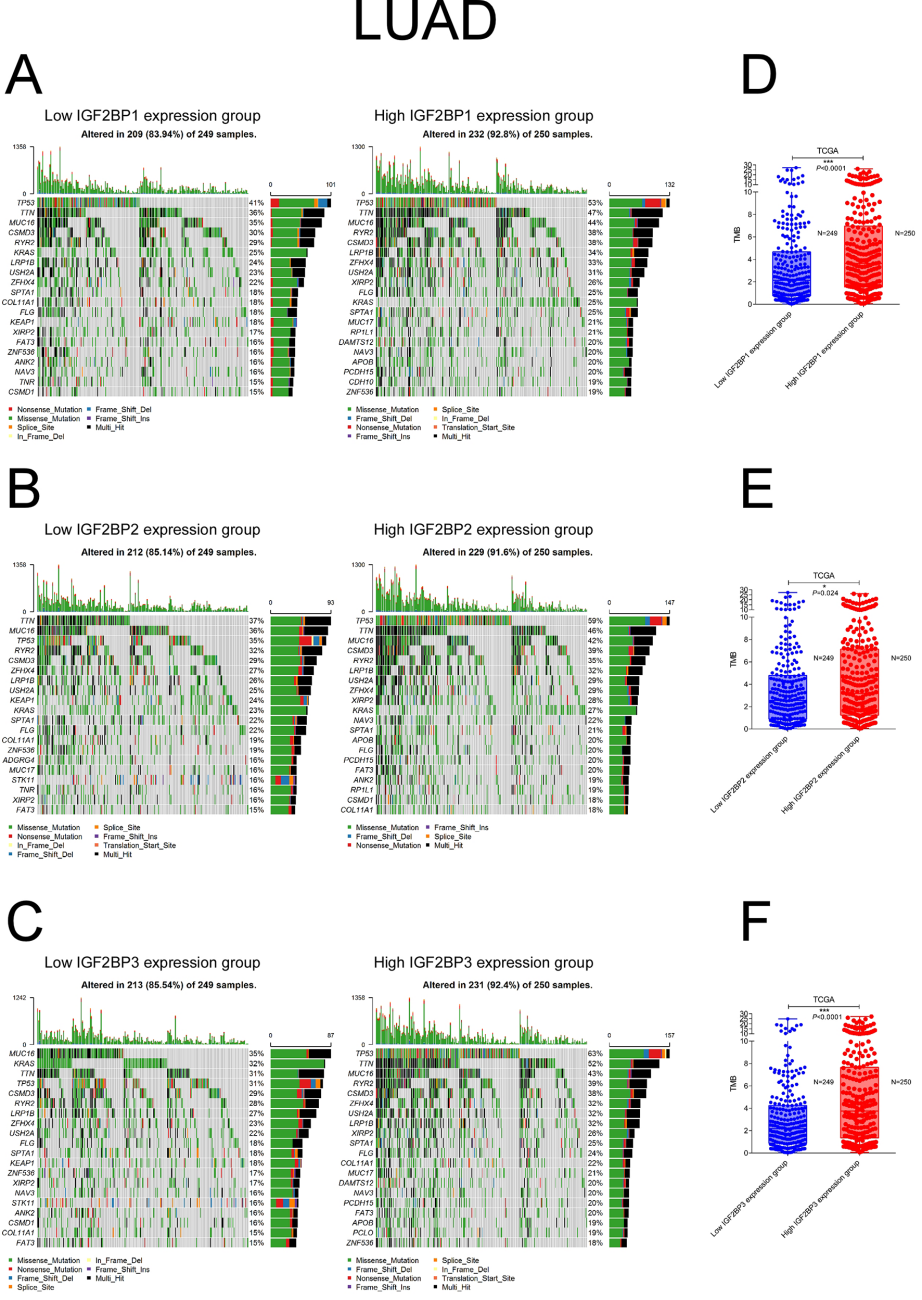
Analysis of mutation burden in different IGF2BPs expression groups in LUAD. Mutation landscape of LUAD tumor samples with low and high IGF2BP1 (**A**), IGF2BP2 (**B**), and IGF2BP3 (**C**) expression. Comparison of total tumor mutation burden of different IGF2BP1 (**D**), IGF2BP2 (**E**), and IGF2BP3 (**F**) expression groups in LUAD. Stars indicate a significant difference between groups. *P < 0.05; **P < 0.01; ***P < 0.001

**Fig. 4:**
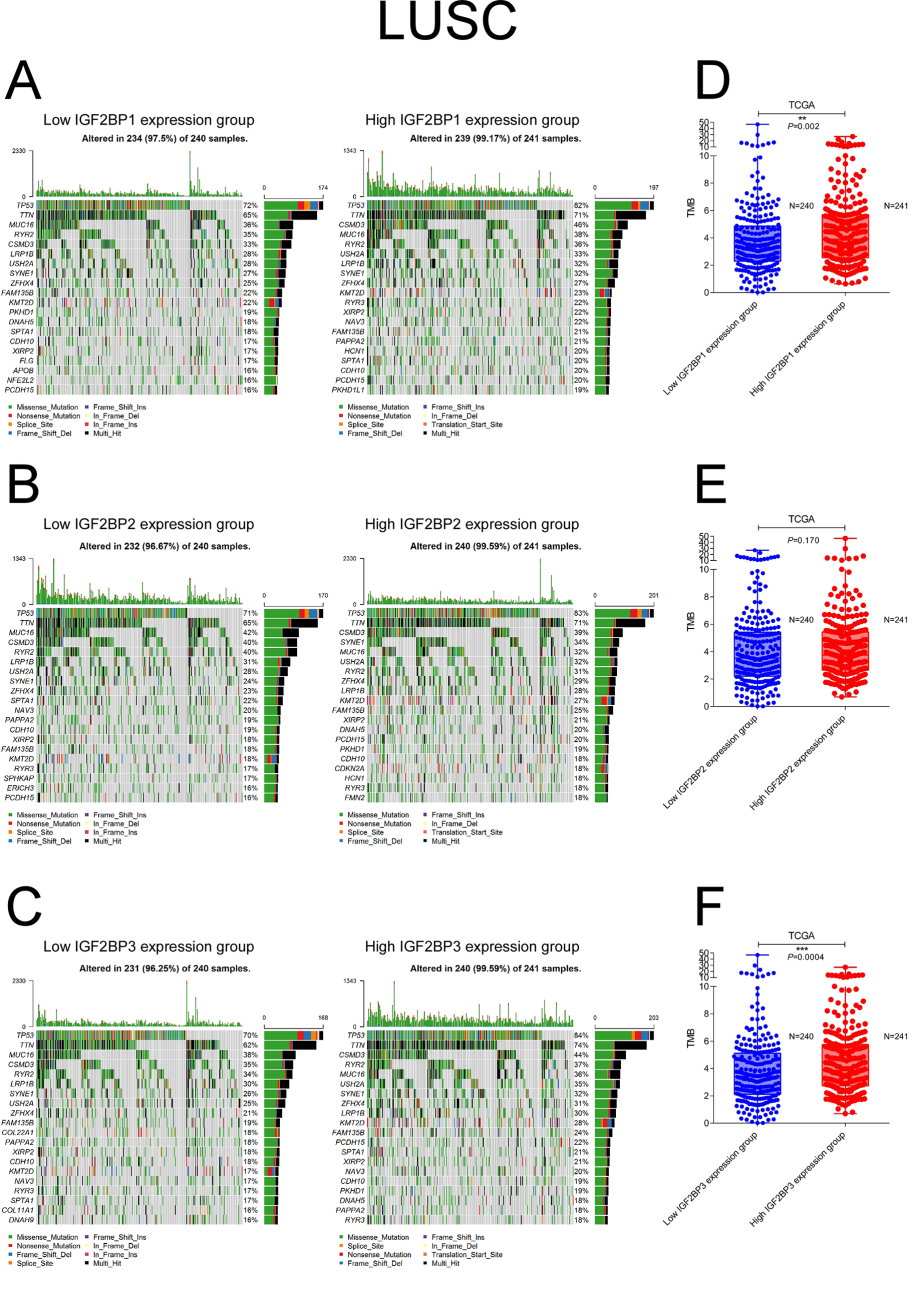
Analysis of mutation burden in different IGF2BPs expression groups in LUSC. Mutation landscape of LUSC tumor samples with low and high IGF2BP1 (**A**), IGF2BP2 (**B**), and IGF2BP3 (**C**) expression. Comparison of total tumor mutation burden of different IGF2BP1 (**D**), IGF2BP2 (**E**), and IGF2BP3 (**F**) expression groups in LUSC. Stars indicate a significant difference between groups. *P < 0.05; **P < 0.01; ***P < 0.001

TMB was evaluated as a biomarker for immunotherapy response in NSCLC. Patients with LUAD and LUSC were categorized into low and high expression groups based on the median expression levels of IGF2BP family genes. We then compared the frequency of gene mutations across these groups. In LUAD, high expression groups of IGF2BP1 and IGF2BP3 demonstrated significantly higher TMBs (IFG2BP1 3.63 vs. 2.14/MB, *P* < 0.0001; IFG2BP3 3.75 vs. 2.18/MB, *P* < 0.0001) (Fig. 3A, C,D, F). LUSC also exhibited similar patterns (IFG2BP1 3.90 vs. 3.33/MB, *P* = 0.002; IFG2BP3 3.86 vs3.42 /MB, *P* = 0.0004) (Fig. 4A, C, D, F). For IGF2BP2, a higher TMB was only observed in the high expression group in LUAD (3.17 vs. 2.56/MB, *P* = 0.024) ( Fig. 3B, E).

In LUAD, groups with high IGF2BP1 expression showed more mutations in SCN1A, NOVA1, BRINP3, RP1L1, BTAF1, TIE1, and CSMD2 compared to the low expression group (Supplemental Fig. 4A). In the high IGF2BP2 expression group, TP53 and RIMS2 mutations were more common, while KEAP1 mutations were less frequent (Supplemental Fig. 4B). Sixteen genes (TP53, TPN, FAM135B, LAMA2, CAD, GRIA4, KRAS, POLE, MYLK, SCN1A, ASTN2, SMARCA4, ITGAX, PIK3R4, CFHR5, BTAF1, and KCNG1) showed mutations that were significantly different between groups with high and low IGF2BP3 expression (Supplemental Fig. 4C). KRAS mutations were more common in the group with low IGF2BP3 expression.

In LUSC, the differences in gene mutations between low and high expression groups of IGF2BP family genes were less pronounced than those observed in LUAD. Specifically, the high expression group of IGF2BP1 in LUSC demonstrated increased mutation frequencies in LIRB3, ZCCHC5, and KLK15 and a decreased frequency in OR4C6 mutations compared to the low expression group (Supplemental Fig. 5A). In the high IGF2BP2 expression group, mutations in TMC3 were more frequent, while mutations in CFHR2 were less common compared to the low expression group (Supplemental Fig. 5B). There were more TP53 and TIMD4 mutations in the IGF2BP3 high expression group than in the IGF2BP3 low expression group in LUSC (Supplemental Fig. 5C).

### Prognostic features of the IGF2BP family in lung cancer patients

**Fig. 5:**
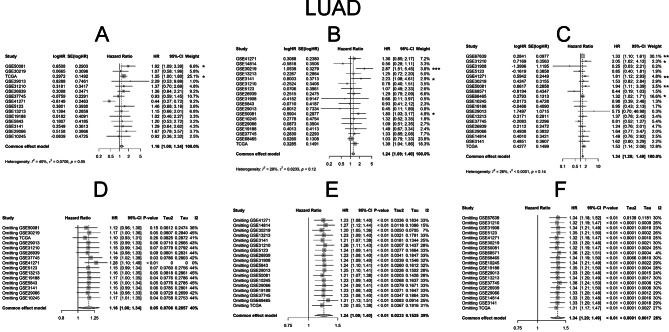
Meta-analyses of prognostic value of IGF2BP family genes in LUAD. Pooled analysis of cox regression analysis between IGF2BP1 (**A**), IGF2BP2 (**B**), IGF2BP3 (**C**) expression and OS in fixed effects models in LUAD patients. Sensitivity analyses through omitting each study evaluating prognostic value of IGF2BP1 (**D**), IGF2BP2 (**E**), IGF2BP3 (**F**)

**Fig. 6:**
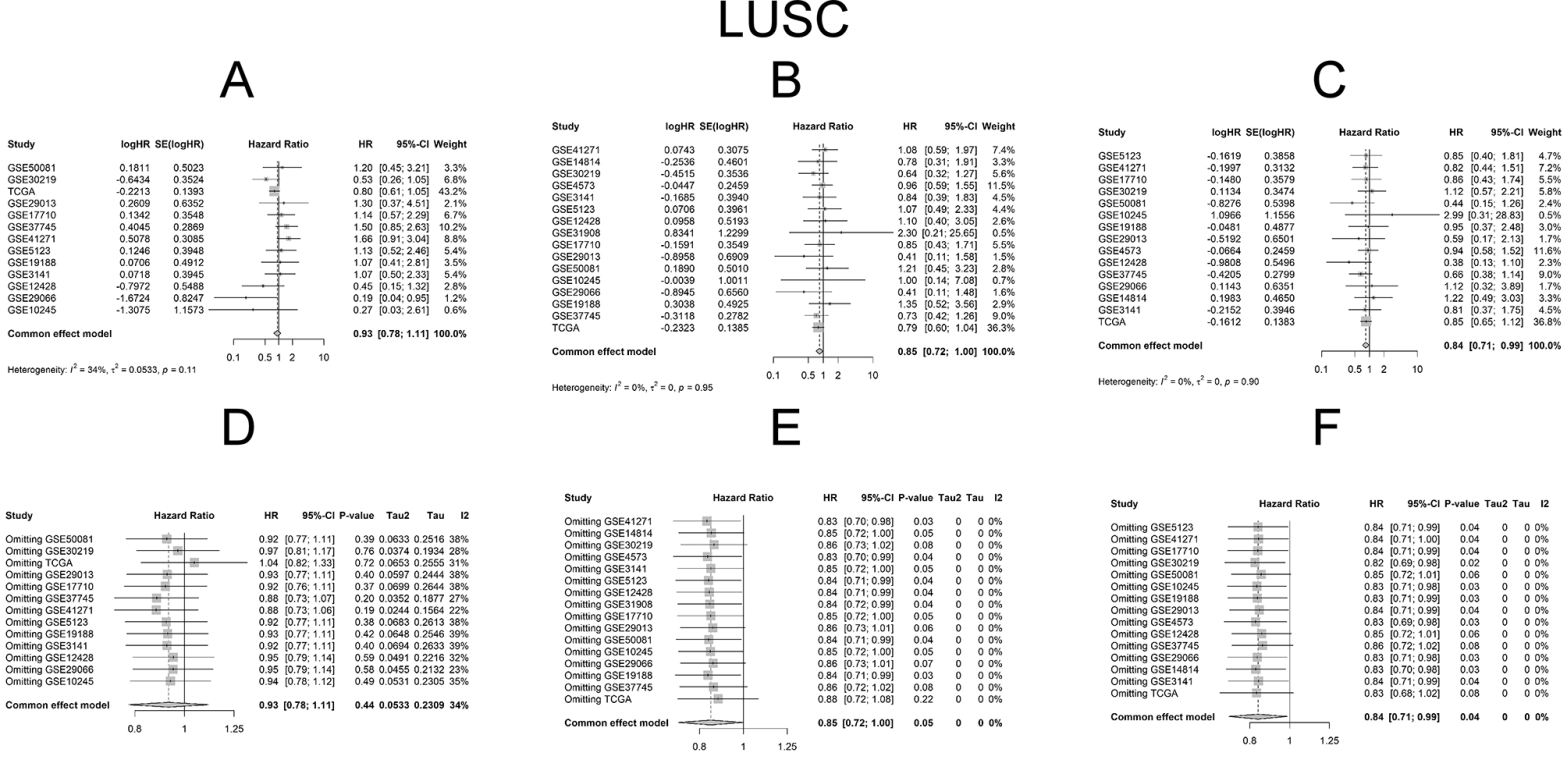
Meta-analyses of prognostic value of IGF2BP family genes in LUSC. Pooled analysis of cox regression analysis between IGF2BP1 (**A**), IGF2BP2 (**B**), IGF2BP3 (**C**) expression and OS in fixed effects models in LUSC patients. Sensitivity analyses through omitting each study evaluating prognostic value of IGF2BP1 (**D**), IGF2BP2 (**E**), IGF2BP3 (**F**)

We conducted a meta-analysis to assess the prognostic values of IGF2BP family genes in lung cancer patients using data from publicly available lung cancer datasets, the OSluca program [36]. Detailed information about those datasets in OSluca program was provide in Supplemental Table 1. Since IGF2BPs was not included in arrays of all datasets and some datasets only include LUAD or LUSC, there were discrepancies between datasets in analyzing processes. Fixed models were used in the analyses because all hypothesis tests for homogeneity were not significant. The results revealed divergent correlations between overall OS and IGF2BP LUSC family genes in LUAD and LUSC. In LUAD patients, OS was negatively correlated with IGF2BP family genes. Conversely, in LUSC patients, IGF2BP2 and IGF2BP3 mRNA expressions were positively correlated with OS. The pooled analysis of OS demonstrated significant differences between different expression groups of IGF2BP1 (HR = 1.160, 95% CI: 1.001–1.343, *P* = 0.047; I^2^ = 40%, *P*_*hom*_ = 0.053, Fig. 5A), IGF2BP2 (HR = 1.236, 95% CI: 1.091–1.400, *P* = 0.001; I^2^ = 29%, *P*_*hom*_ = 0.119, Fig. 5B) and IGF2BP3 (HR = 1.338, 95% CI: 1.204–1.456, *P* < 0.0001; I^2^ = 26%, *P*_*hom*_ = 0.145, Fig. 5C) in LUAD, with slight heterogeneities observed in fixed effects models. For LUSC, IGF2BP2 (HR = 0.847, 95% CI: 0.719–0.997, *P* = 0.047; I^2^ = 0%, *P*_*hom*_ = 0.955, Fig. 6B) and IGF2BP3 (HR = 0.838, 95% CI: 0.711–0.988, *P* = 0.035; I^2^ = 0%, *P*_*hom*_ = 0.903, Fig. 6C) also exhibited minimal heterogeneity in fixed effects models.

To assess the individual study’s impact on the pooled estimate, sensitivity analyses were performed. This involved excluding one study at a time and recalculating the pooled HR estimates for the remaining studies. There were no significant differences between the groups for IGF2BP1 (Fig. 5D), IGF2BP2 (Fig. 5E) and IGF2BP3 (Fig. 5F) in LUAD. The similar results were observed for IGF2BP2 (Fig. 6E) and IGF2BP3 (Fig. 6F) in LUSC, affirming the reliability of our results.

Publication bias was assessed using funnel and radial plots. These plots did not reveal any significant publication bias for the HR of OS (Supplemental Fig. 6 and Supplemental Fig. 7), suggesting low publication bias in this meta-analysis.

We further investigated whether IGF2BP family genes have varying survival effects in LUAD patients with or without EGFR-sensitive mutations, considering the distinct treatment responses and OS observed in these subgroups. This analysis was conducted using TCGA LUAD samples with comprehensive mutation data. No significant differences in OS were observed between high and low mRNA expression groups of IGF2BP family genes in LUAD patients, regardless of their EGFR mutation status (Supplemental Fig. 8). Table 2 detailed the analysis of heterogeneities in the correlation between IGF2BPs mRNA expression and OS among LUAD patients with or without EGFR-sensitive mutations. The analysis indicated no significant heterogeneities, suggesting that the impact of IGF2BP family gene expression on OS is consistent across LUAD patients, irrespective of their EGFR mutation status.

**Table 2:** Associations of IGF2BPs with OS in LUAD patients with or without EGFR mutations

Variables	IGF2BP1 (Event/No.)		Overall survival (OS)			IGF2BP2 (Event/No.)		Overall survival (OS)			IGF2BP3 (Event/No.)		Overall survival (OS)		
	Low	High	HR (95% CI)	*P* ^a^	*P*_hom_	Low	High	HR (95% CI)	*P* ^a^	*P*_hom_	Low	High	HR (95% CI)	*P* ^a^	*P*_hom_
EGFR mutations															
No	8/28	12/28	1.17 (0.45–3.03)	0.75		9/28	11/28	1.13 (0.47–2.73)	0.785		9/28	11/28	1.16 (0.48–2.83)	0.738	
Yes	2/12	3/11	1.14 (0.16–8.15)	0.900	0.981	4/12	1/11	0.23 (0.000004–135.57)	0.394	0.720	3/12	2/11	0.39 (0.04–3.78)	0.415	0.382

Abbreviations: LUAD, lung adenocarcinoma; CI, confidence interval; HR, hazards ratio; hom, heterogeneity test. The results were in **bold**, if *P* < 0.05

### Genetic mutations in the IGF2BP family and their associations with OS and DFS for NSCLC patients

Epigenetic alteration plays a crucial role in early malignancies [38]. Therefore, we assessed the role of epigenetic alterations, with a focus on the IGF2BP family genes and their association with OS and DFS in LUAD and LUSC. The analysis utilized data from cBioPortal for LUAD and LUSC (TCGA, Firehose Legacy). Our findings revealed that alterations in IGF2BP family genes occurred in 57 out of 507 LUAD patients (11.2%) and 180 out of 496 LUSC patients (36.3%). The mutation rates for IGF2BP1, IGF2BP2, and IGF2BP3 in LUAD were 3%, 4%, and 5% respectively (Supplemental Fig. 9A). The mutation rates for these genes in LUSC were 2.8%, 35%, and 3% respectively (Supplemental Fig. 9E).

Further analysis was conducted on the relationship between IGF2BP family members based on their mRNA expression (RNA Seq V2 RSEM), using Pearson’s correlation. The results revealed a positive correlation between IGF2BP3 and IGF2BP1 (*P* = 0.003) and IGF2BP2 (*P* < 0.001) in LUAD (Supplemental Fig. 9B). In LUSC, a positive correlation was observed between IGF2BP1 and IGF2BP2 (*P* = 0.008) (Supplemental Fig. 9F).

Additionally, we examined the correlation between genetic alterations in the IGF2BP family and OS and DFS in lung cancer patients. Kaplan-Meier plots and log-rank tests were performed. The results indicated that genetic alterations in the IGF2BP family genes were not significantly correlated with OS and DFS in both LUAD and LUSC (Supplemental Fig. 9C, D, G, H).

### GO and KEGG Enrichment Analysis of IGF2BP Family in NSCLC patients

**Fig. 7:**
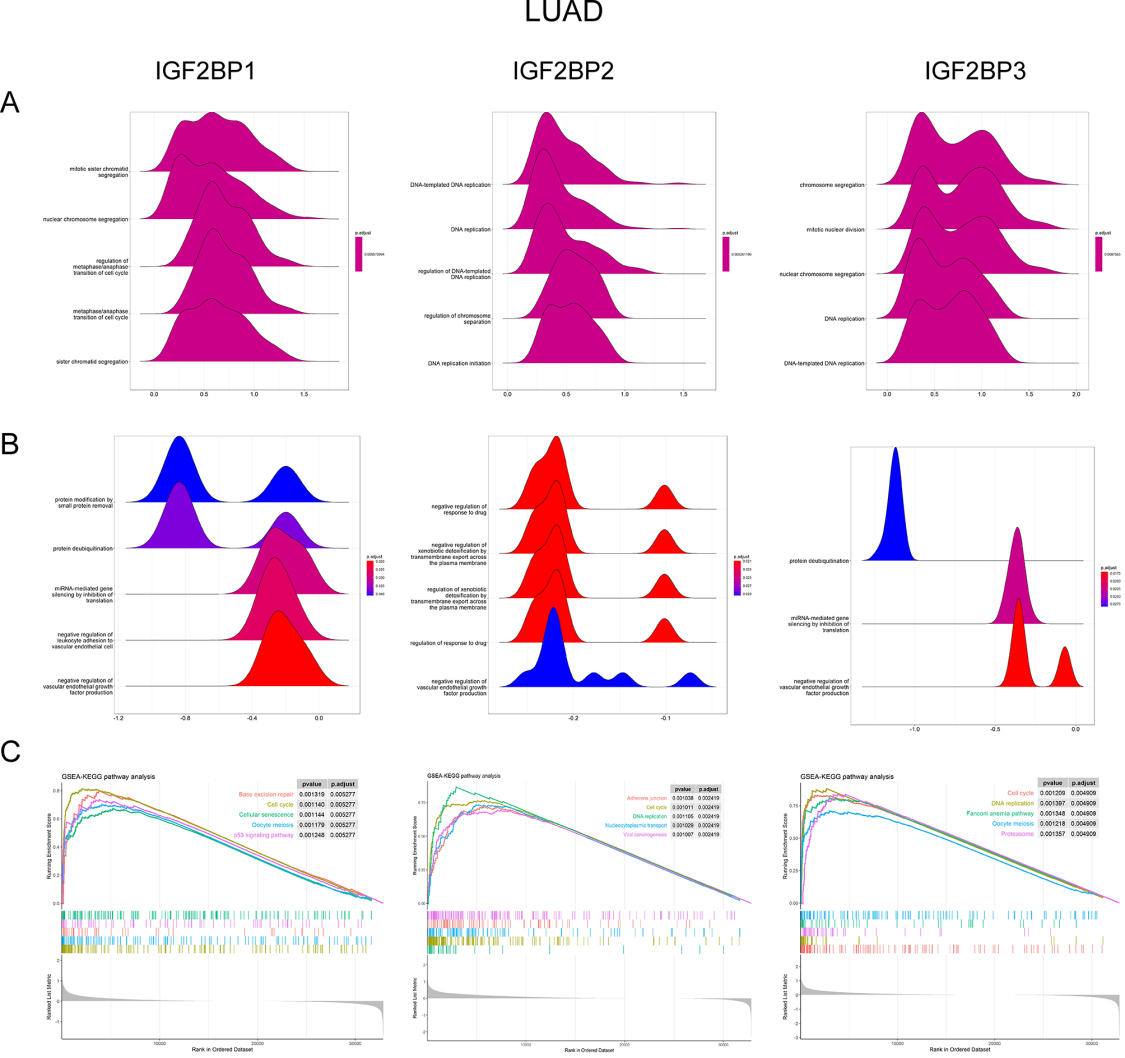
Gene Set Enrichment Analysis of GO and KEGG pathways in LUAD between different IGF2BPs expression groups. (**A**) Results of GO functions positively associated with IGF2BP1, IGF2BP2 and IGF2BP3 in LUAD. (**B**) Results of GO functions negatively associated with IGF2BP1, IGF2BP2 and IGF2BP3 in LUAD. (**C**) Results of KEGG pathways between different IGF2BPs expression groups in LUAD

**Fig. 8:**
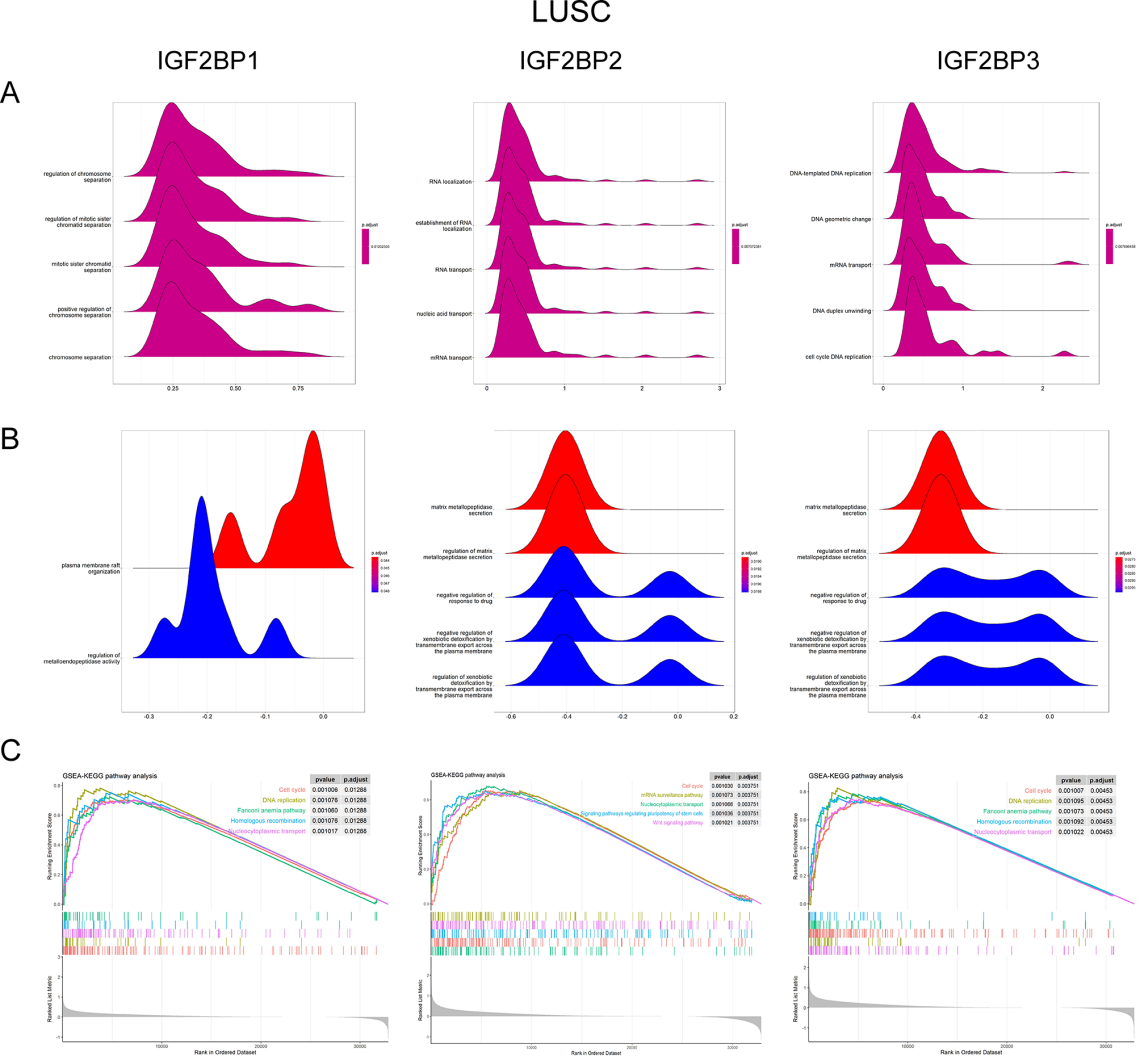
Gene Set Enrichment Analysis of GO and KEGG pathways in LUSC between different IGF2BPs expression groups. (**A**) Results of GO functions positively associated with IGF2BP1, IGF2BP2 and IGF2BP3 in LUSC. (**B**) Results of GO functions negatively associated with IGF2BP1, IGF2BP2 and IGF2BP3 in LUSC. (**C**) Results of KEGG pathways between different IGF2BPs expression groups in LUSC

Acknowledging the contrasting features of IGF2BP2 and IGF2BP3 between LUAD and LUSC, we utilized GSEA to elucidate underlying biological functions and signaling pathways. The GO analysis indicated a positive association of cell cycle, DNA replication, and chromosome segregation processes with all IGF2BP family genes in LUAD (Fig. 7A). Similar trends were noted for IGF2BP1 and IGF2BP3 in LUSC, with RNA transport and localization being upregulated in the IGF2BP2 high expression group (Fig. 8A). Additionally, the negative regulation of VEGF production inversely correlated with IGF2BP genes in LUAD (Fig. 7B), suggesting that high expression of these genes might accelerate cancer progression via enhanced VEGF production, potentially explaining the poorer overall survival in LUAD patients with high IGF2BP expression. In LUSC, processes related to matrix metallopeptidase, crucial in cancer invasion and metastasis, were negatively correlated with IGF2BP genes (Fig. 8B), which may account for the better overall survival in LUSC patients with high IGF2BP expression. The KEGG analysis revealed the upregulation of the cell cycle pathway in high expression groups of all three IGF2BP family genes in both LUAD and LUSC (Figs. 7C and 8C), yet no downregulated KEGG pathways were identified in these groups.

### IGF2BP family genes and chemotherapy sensitivity

**Fig. 9:**
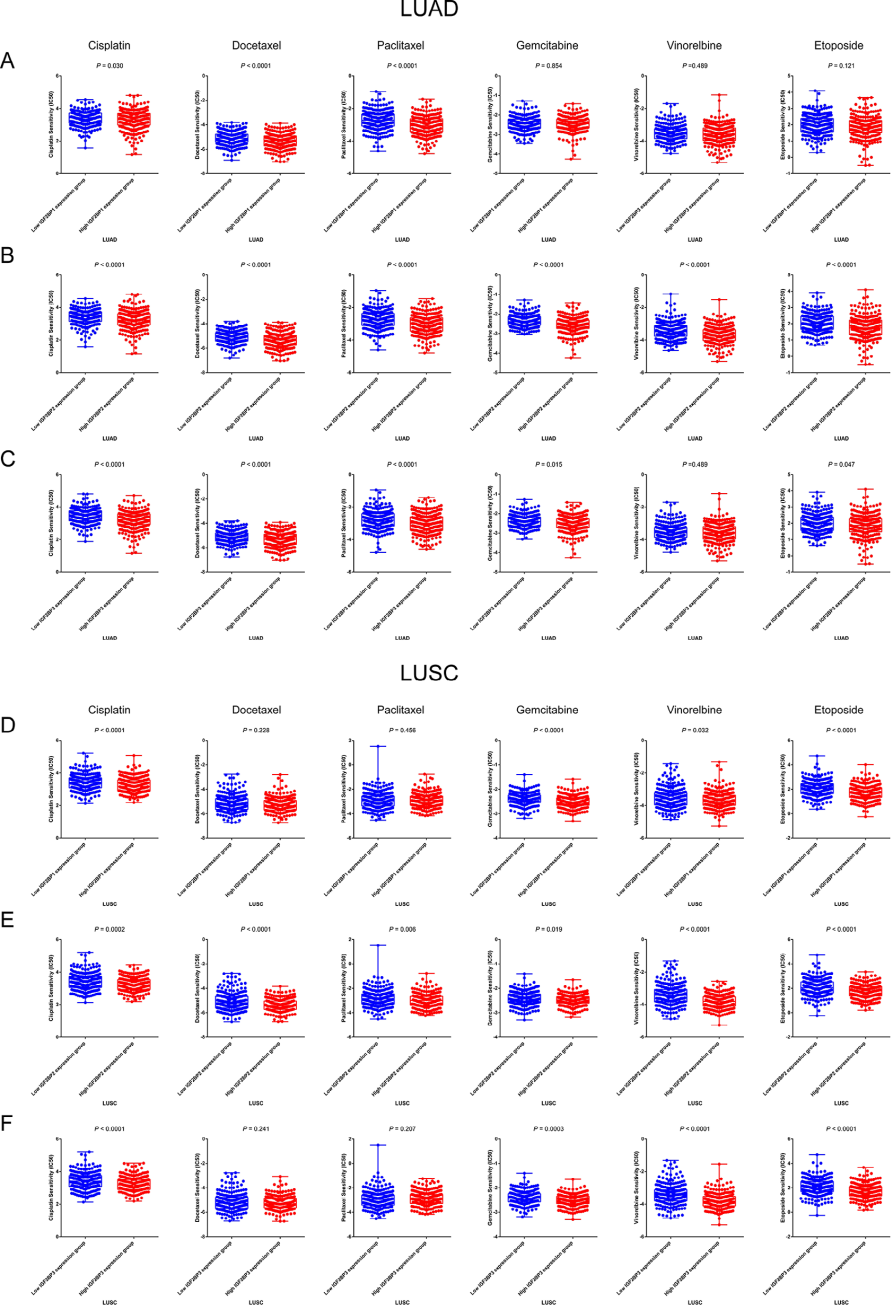
IGF2BPs in the role of chemotherapy in NSCLC. The correlation between different IGF2BP1 (**A**), IGF2BP2 (**B**), IGF2BP3 (**C**) mRNA expression groups and estimated IC50 value of cisplatin, doxorubicin, paclitaxel, gemcitabine, vinorelbine and etoposide in LUAD from TCGA dataset. The correlation between different IGF2BP1 (**D**), IGF2BP2 (**E**), IGF2BP3 (**F**) mRNA expression groups and estimated IC50 value of cisplatin, doxorubicin, paclitaxel, gemcitabine, vinorelbine and etoposide in LUSC from TCGA dataset. Wilcoxon signed-rank tests were used to compare the estimated IC50 value of chemotherapy between groups

Given the established association between IGF2BP family genes and prognosis, we further explored their relationship with chemotherapy sensitivity. Using the “pRRophetic” R package, we calculated the IC50 to predict the response to various chemotherapy drugs (cisplatin, docetaxel, paclitaxel, gemcitabine, vinorelbine, and etoposide). The IGF2BP2 high expression group in LUAD demonstrated increased sensitivity to all six chemotherapy agents (Fig. 9B) (*P*_*adjust*_ < 0.0001 for each). A similar trend, barring gemcitabine, was observed in LUSC (Fig. 9E). IGF2BP1 exhibited a positive correlation with sensitivity to docetaxel (*P*_*adjust*_ < 0.0001) and paclitaxel (*P*_*adjust*_ < 0.0001) in LUAD (Fig. 9A) and to cisplatin (*P*_*adjust*_ < 0.0001), gemcitabine (*P*_*adjust*_ < 0.0001), and etoposide (*P*_*adjust*_ < 0.0001) in LUSC (Fig. 9D). Additionally, IGF2BP3’s high expression group in LUAD showed lower IC50 values for cisplatin (*P*_*adjust*_ < 0.0001), docetaxel (*P*_*adjust*_ < 0.0001) and paclitaxel (*P*_*adjust*_ < 0.0001) (Fig. 9C), while in LUSC, this group exhibited lower IC50 values for cisplatin (*P*_*adjust*_ < 0.0001), gemcitabine (*P*_*adjust*_ = 0.009), vinorelbine (*P*_*adjust*_ < 0.0001), and etoposide (*P*_*adjust*_ < 0.0001) (Fig. 9F). These findings indicated a potential role for IGF2BP family genes on chemotherapy efficacy.

## Discussion

The highly conserved proteins IGF2BP1, IGF2BP2, and IGF2BP3 are members of the IGF2BP family, which is recognized for its RNA-binding capabilities that affect the fate of the transcript targets it binds. These proteins exhibit a 56% amino acid sequence identity, with a higher degree of similarity within their protein domains, suggesting shared biochemical functions [11]. IGF2BPs play crucial roles in the progression, metastasis, prognosis, and cancer immunity of several human cancers [39, 40], including NSCLC [41]. IGF2BP1, identified as an m6A reader, has been shown to stabilize TK1 [42], BUB1 [43], and SIK2 [44], contributing to the malignant behavior of NSCLC. Furthermore, it engages with CDCA4 to modulate the proliferation of LUAD through the PI3K/AKT signaling pathway [45]. This interaction is also implicated in the IGF2BP1/Netrin-1 axis, which plays an oncogenic role in high glucose-treated NSCLC cells [46]. In addition, Zhu et al. identified a positive feedback loop, c-Myc/MNX1-AS1/IGF2BP1, which was found to accelerate cell-cycle progression and enhance the sustained proliferation of lung cancer cells [47]. IGF2BP2 is known to stabilize the lncRNA MALAT1, enhance ATG2 expression, and promote NSCLC proliferation [48]. Zhu et al. noted that IGF2BP2 stabilizes TGFBR1, accelerating NSCLC stemness [49]. Additionally, the IGF2BP2/LATS1 axis has been found to promote the growth of lung adenocarcinoma [50]. Regarding IGF2BP3, recent studies indicate its role in regulating metabolic reprogramming and promoting resistance to EGFR inhibitors in NSCLC [51]. IGF2BP3 overexpression stabilizes anti-ferroptotic factors like SLC3A2 and ACSL3, inhibiting ferroptosis in lung adenocarcinoma cells [52]. Furthermore, IGF2BP3 contributes to lung adenocarcinoma progression by modulating the PI3K/AKT signaling pathway [53] and promoting partial EMT and metastasis through the MCM5/Notch axis [54]. Targeting IGF2BPs has been suggested in several studies as a strategy to inhibit malignant behaviors of cancers in vitro [55–58].

Despite the extensive research on IGF2BP family genes, there has been a lack of studies specifically addressing these genes in LUSC, a cancer type with distinctly different biological behaviors compared to LUAD. To address this gap, we analyzed the expression, mutation, immune involvement, resistance to thermotherapy, and prognostic roles of different IGF2BP family members in LUSC, with comparative insights from LUAD. Our findings revealed that mRNA expression levels of IGF2BP1 and 3 were significantly upregulated in both LUAD and LUSC tissues. This observation was corroborated by analyses of our own collected NSCLC samples. Furthermore, immunohistochemical analysis conducted on our samples indicated potential overexpression of IGF2BP family genes in both LUSC and LUAD compared to healthy lung tissues. These results align with previous studies [48] and suggest that IGF2BPs may play significant roles in the pathogenesis of LUSC as well. However, there were also inconsistent results between analyses of TCGA datasets and of our own collected NSCLC samples. TCGA datasets showed that IGF2BPs mRNA levels in LUSC were higher than those in LUAD, which was not validated by our own samples. In addition, we observed IGF2BP2 mRNA levels in LUAD were higher compared to normal lung tissues, but TCGA samples showed that there was not significantly different in IGF2BP2 mRNA expression levels between LUAD and adjacent normal lung tissues. We tried to explain those difference using a power analysis by PASS software (version 11, NCSS Data Network, Inc. New York, NY, USA) and found that we only get the power of 0.385 to find the mean difference of 1 at the conventional 0.05 alpha error probability by our sample size. That is to say, those inconsistent results above probably owing to our limited sample size and need to be determined by large sample size evaluation in the future.

Immunotherapy has been a transformative approach to treating various solid malignant tumors, including NSCLC. Recent research has shown that the IGF2BP family genes play significant roles in regulating the tumor microenvironment [39], immune evasion [31, 59], and anti-tumor immunity [10]. To delve deeper into these aspects in LUAD and LUSC, we investigated the association between IGF2BP1/2/3 and cancer immunity using the TCGA dataset. The result revealed that IGF2BP2 mRNA expression levels correlate with the infiltration levels of several immune cells and the expression of several immunotherapy target genes in both LUAD and LUSC. However, the relationship between IGF2BP2 and those immune factors were opposite between LUAD and LUSC. The copy number variations of IGF2BP2 were linked with diverse immune cell infiltration levels in LUSC. Significantly higher TMBs were observed in high-expression groups of IGF2BP1, IGF2BP2, and IGF2BP3 in LUAD. In contrast, in LUSC, only high-expression groups of IGF2BP1 and IGF2BP3 showed similar trends. We also noted more gene mutations in different IGF2BP1/2/3 expression groups in LUAD compared to LUSC. This suggests a more complex interaction between IGF2BP family genes and cancer genomics in NSCLC.

Previous research has established a correlation between IGF2BP1, IGF2BP2, and IGF2BP3 and poor prognosis in NSCLC patients [41, 48]. To delve deeper, we conducted a meta-analysis to compare the prognostic value of the IGF2BP family genes in LUAD and LUSC patients. The results showed that OS in LUAD patients is negatively correlated with IGF2BP family genes, consistent with previous findings. In contrast, LUSC patients exhibited a positive correlation between IGF2BP2 and IGF2BP3 mRNA expression and OS. We also observed that the survival differences attributed to IGF2BP family genes in LUAD patients were not significantly affected by the presence or absence of EGFR-sensitive mutations. This suggests that EGFR mutation pathways do not influence the biological functions of IGF2BP family genes in LUAD. Additionally, no association was found between genetic mutations in IGF2BP family genes and OS in NSCLC patients. GSEA revealed that expression of genes that involve to cell cycle, DNA replication, and chromosome segregation processes were positively associated with IGF2BP family genes in both LUAD and LUSC. VEGF production was inversely associated with IGF2BP family genes in LUAD. Conversely, biological processes related to matrix metallopeptidase were negatively correlated with IGF2BP family genes in LUSC. These results suggest that high expression of IGF2BP1/2/3 may promote VEGF production, accelerating cancer progression in LUAD. On the other hand, high expression of IGF2BPs in LUSC might inhibit matrix metallopeptidase activity, thereby preventing invasion and metastasis. This hypothesis could partly explain the observed survival differences between LUAD and LUSC patients with high IGF2BP expression. However, further in vitro and in vivo studies are necessary to substantiate these findings.

To determine the role of IGF2BPs in chemotherapy resistance in NSCLC, we examined the correlation between their expression and the IC50 of six common chemotherapy drugs (cisplatin, docetaxel, paclitaxel, gemcitabine, vinorelbine and etoposide) used in NSCLC treatment. Our findings suggest that docetaxel and paclitaxel exhibit significantly lower IC50 in high IGF2BPs expression groups in LUAD, while cisplatin and etoposide exhibit significantly lower IC50 in high IGF2BPs expression groups in LUSC. These results align with GSEA results, which revealed that tumors with high IGF2BP family gene expression showed activated cell cycle, DNA replication, and chromosome segregation processes, thereby enhancing sensitivity to several chemotherapy drugs. Recent study also reported IGF2BPs overexpression lead to docetaxel chemosensitivity enhancement in advanced prostate cancer [60], which is consistent with our results. However, there was also studies indicating that knockdown of IGF2BP3 increase the platinum sensitivity, but not taxol sensitivity in ovarian cancer cells [61]. Those phenomenon may due to different tumor background.

It is important to recognize the potential limitations of our study. Firstly, this was a retrospective analysis primarily based on public datasets, and our findings necessitate validation in larger, prospective studies. Secondly, the mechanisms through which IGF2BPs confer survival benefits in LUSC remain incompletely understood. Whether the negative regulation of matrix metallopeptidase, which could potentially inhibit LUSC invasion and metastasis, plays a role in this phenomenon needs to be further investigated through experimental verification in future studies.

## Conclusion

In this study, we investigated the expression and prognostic significance of the IGF2BP family in NSCLC. This research contributes to a deeper understanding of the molecular heterogeneity and complexity in LUSC and LUAD, paving the way for novel approaches in the diagnosis and treatment of NSCLC. Our findings also demonstrate that overexpression of IGF2BP2 is significantly associated with cancer immunity in NSCLC patients. Interestingly, mutations in the IGF2BP family did not result in significant differences in OS or DFS in LUAD and LUSC patients. Moreover, higher mRNA expressions of IGF2BP2 and IGF2BP3 were positively correlated with OS in LUSC, yet showed a negative association with OS in LUAD. GSEA indicated that matrix metallopeptidase activity and VEGF production might be involved in these differential outcomes. These insights suggest that IGF2BP1, IGF2BP2, and IGF2BP3 may have distinct roles in LUAD and LUSC. Therefore, any new therapeutic strategy targeting the IGF2BP family genes in NSCLC must be approached with caution, especially considering the varied mechanisms at play in different histological subtypes. Further research is required to fully elucidate these mechanisms.

### Electronic supplementary material

Below is the link to the electronic supplementary material.

Supplementary Material 1

## Data Availability

The datasets utilized in this research are accessible to the public. The resources are accessible via the TCGA portal at [https://portal.gdc.cancer.gov] and the UCSC Xena website’s GDC hub at [http://xena.ucsc.edu/public]. Data from the samples collected for this study are available upon request.

## Abbreviations

NSCLC: Non-small cell lung cancer
IGF2BP: Insulin-like growth factor-2 mRNA-binding protein
TIMER: Tumor IMmune Estimation Resource
UALCAN: University of ALabama at Birmingham CANcer data analysis Portal
TCGA: The Cancer Genome Atlas
OS: Overall survival
DFS: Disease-free survival
LUAD: Lung adenocarcinoma
LUSC: Lung squamous cell carcinoma
GO: Gene Ontology
KEGG: Kyoto Encyclopedia of Genes and Genomes
