# Supplementary material for: Systematic analysis of IGF2BP family members in non-small-cell lung cancer
Source: Hum Genomics. 2024 Jun 12;18:63. doi: 10.1186/s40246-024-00632-6 (PMC11167947; doi:10.1186/s40246-024-00632-6)
Supplement: Supplementary file 1 — Supplementary Material 1 [file 40246_2024_632_MOESM1_ESM.docx]

**Supplementary description**


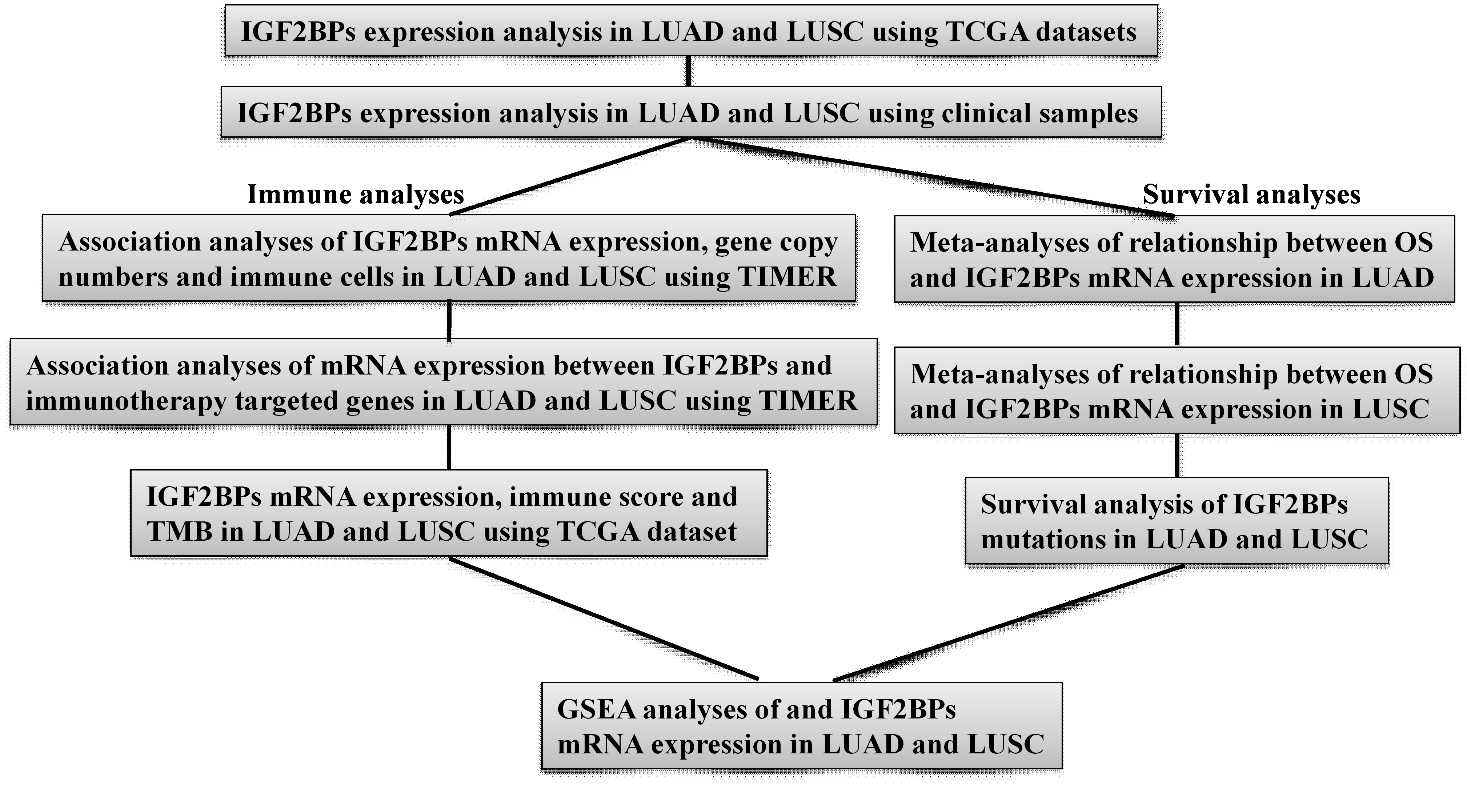
 **Supplemental Figure 1**. A schematic diagram of entire study analysis process.


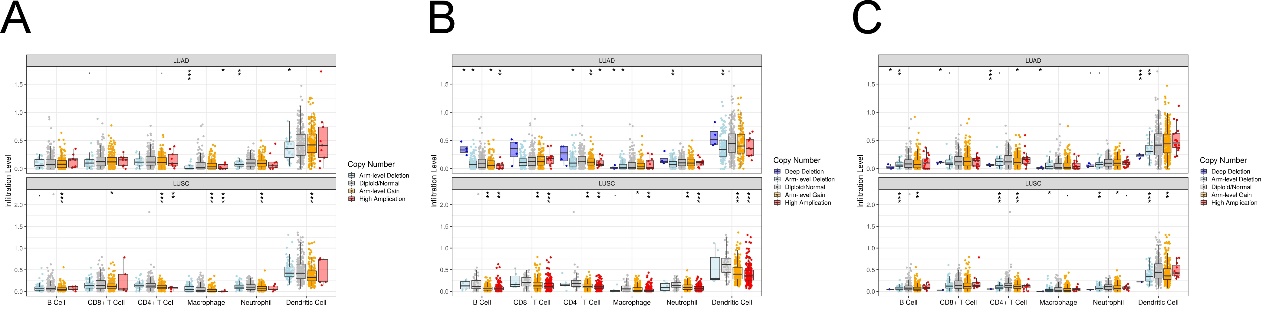


**Supplemental Figure 2**. Correlation of IGF2BP1/2/3gene copy numbers with immune infiltration levels in LUAD and LUSC. Association between IGF2BP1 (A), IGF2BP2 (B) IGF2BP3 (C) copy numbers and immune cell infiltration levels in NSCLC cohorts from TCGA database.**P* < 0.05; ***P* < 0.01; ****P* < 0.001.


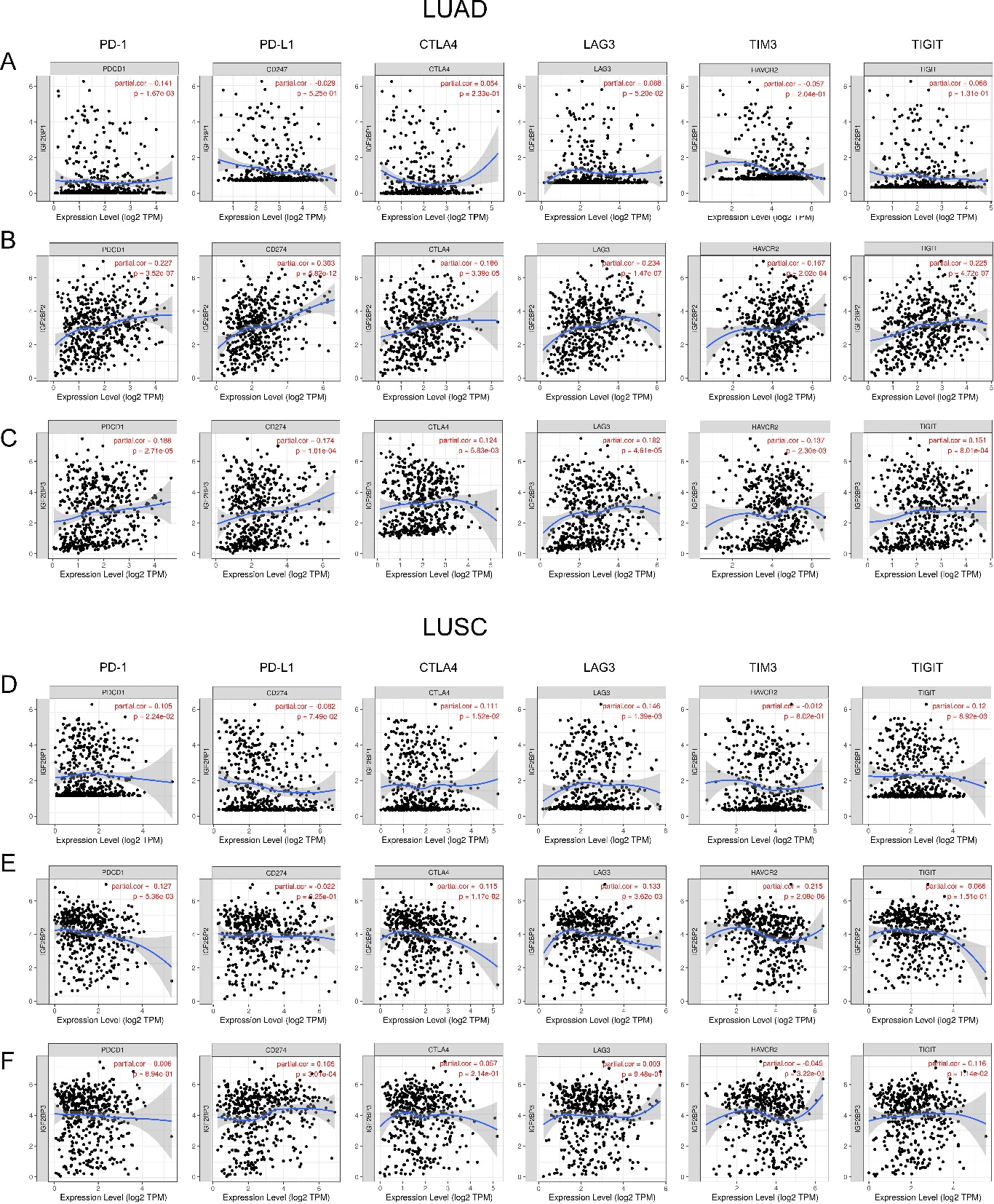


**Supplemental Figure 3**. Correlation analysis of the expression of six key immune pathway genes and IGF2BPs expression in NSCLC. The association of expression of PD-1, PD-L1, CTLA4, LAG-3, TIM-3 and TIGIT with IGF2BP1 (A), IGF2BP2 (B) and IGF2BP3 (C) in LUAD. The association of expression of PD-1, PD-L1, CTLA4, LAG-3, TIM-3 and TIGIT with IGF2BP1 (D), IGF2BP2 (E) and IGF2BP3 (F) in LUSC.


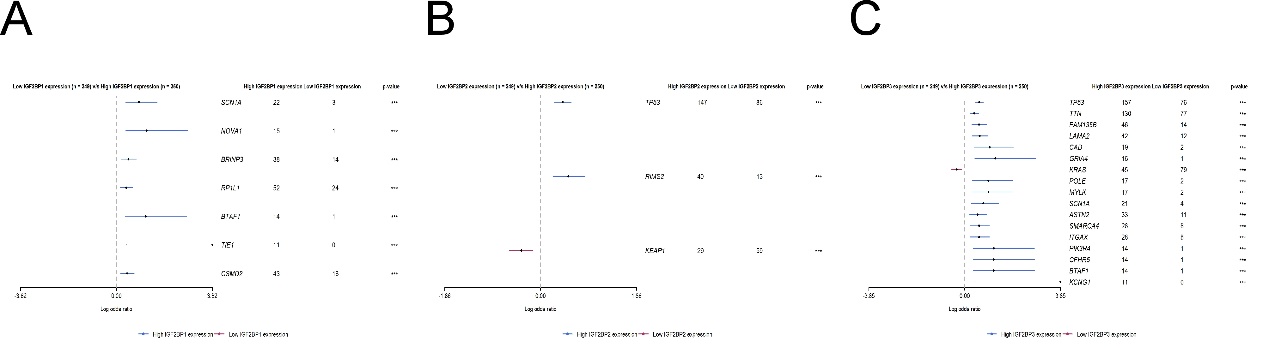


**Supplemental Figure 4**. Comparison of prevalent mutation frequencies between different IGF2BPs expression groups in LUAD. (A) Comparison of prevalent mutation frequencies between low and high IGF2BP1 expression groups in LUAD. (B) Comparison of prevalent mutation frequencies between low and high IGF2BP2 expression groups in LUAD. (C) Comparison of prevalent mutation frequencies between low and high IGF2BP3 expression groups in LUAD. Stars indicate a significant difference between groups. **P* < 0.05; ***P* < 0.01; ****P* < 0.001.


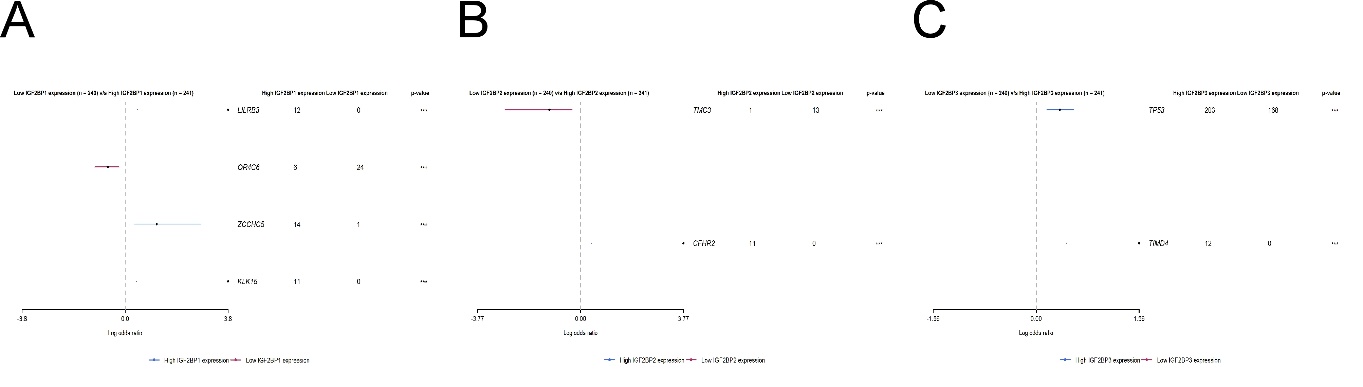


**Supplemental Figure 5**. Comparison of prevalent mutation frequencies between different IGF2BPs expression groups in LUSC. (A) Comparison of prevalent mutation frequencies between low and high IGF2BP1 expression groups in LUSC. (B) Comparison of prevalent mutation frequencies between low and high IGF2BP2 expression groups in LUSC. (C) Comparison of prevalent mutation frequencies between low and high IGF2BP3 expression groups in LUSC. Stars indicate a significant difference between groups. **P* < 0.05; ***P* < 0.01; ****P* < 0.001.


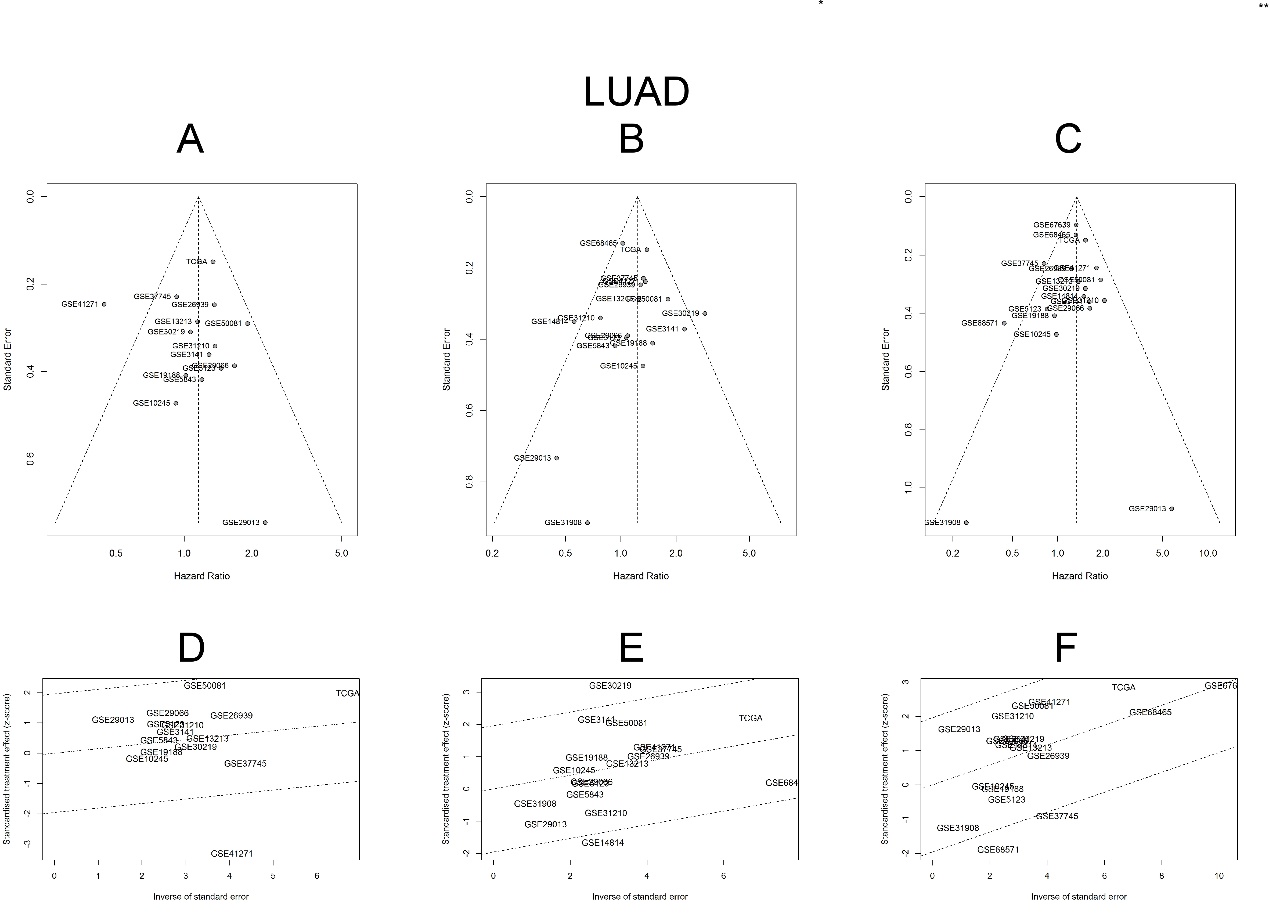


**Supplemental Figure 6**. Funnel and radial plots of IGF2BPs prognostic value meta-analyses in LUAD. Funnel plots showed the publication bias of the individual studies evaluating prognostic value of IGF2BP1 (A), IGF2BP2 (B), IGF2BP3 (C). Radial plots of the individual studies evaluating prognostic value of IGF2BP1 (D), IGF2BP2 (E), IGF2BP3 (F).


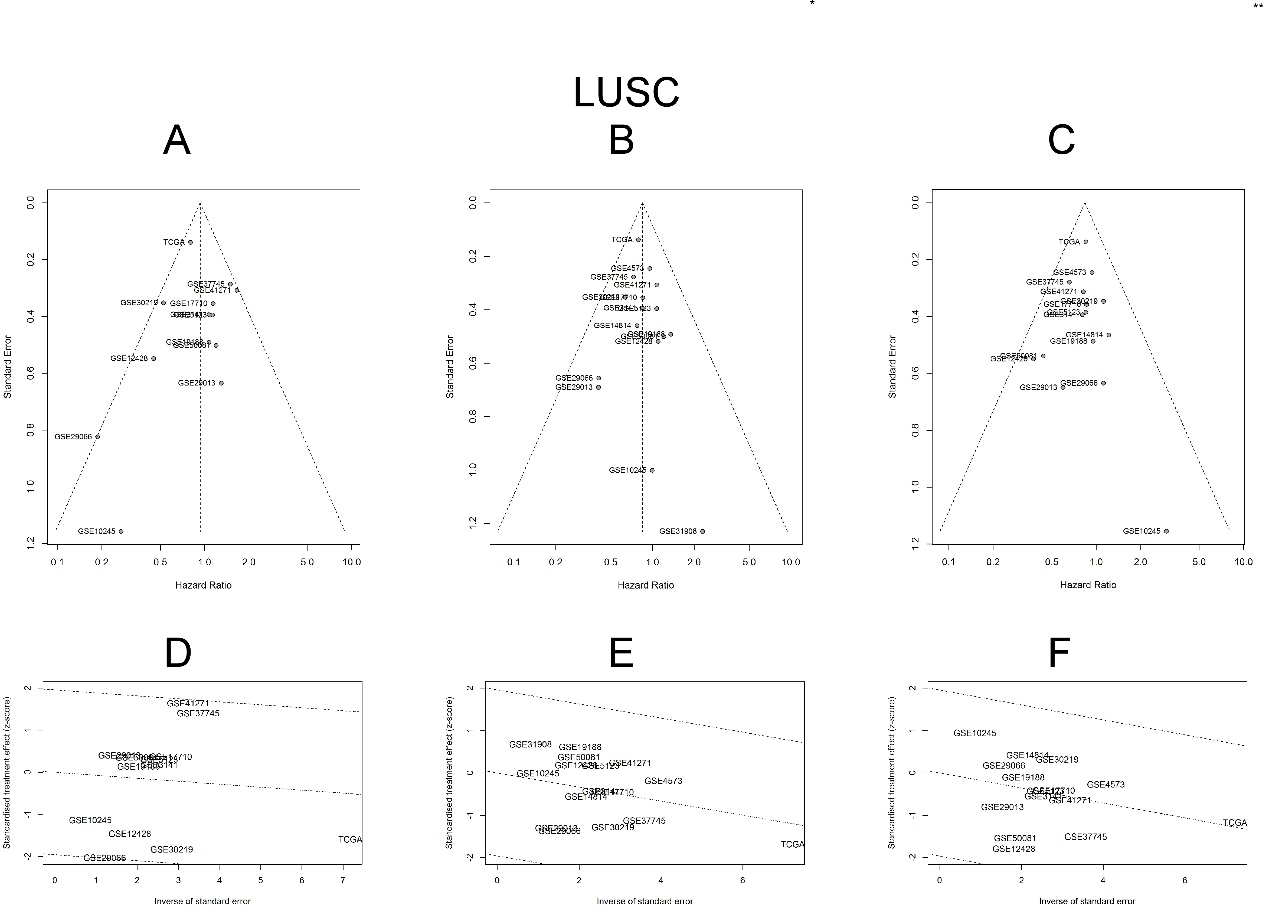


**Supplemental Figure 7**. Funnel and radial plots of IGF2BPs prognostic value meta-analyses in LUSC. Funnel plots showed the publication bias of the individual studies evaluating prognostic value of IGF2BP1 (A), IGF2BP2 (B), IGF2BP3 (C). Radial plots of the individual studies evaluating prognostic value of IGF2BP1 (D), IGF2BP2 (E), IGF2BP3 (F).

**
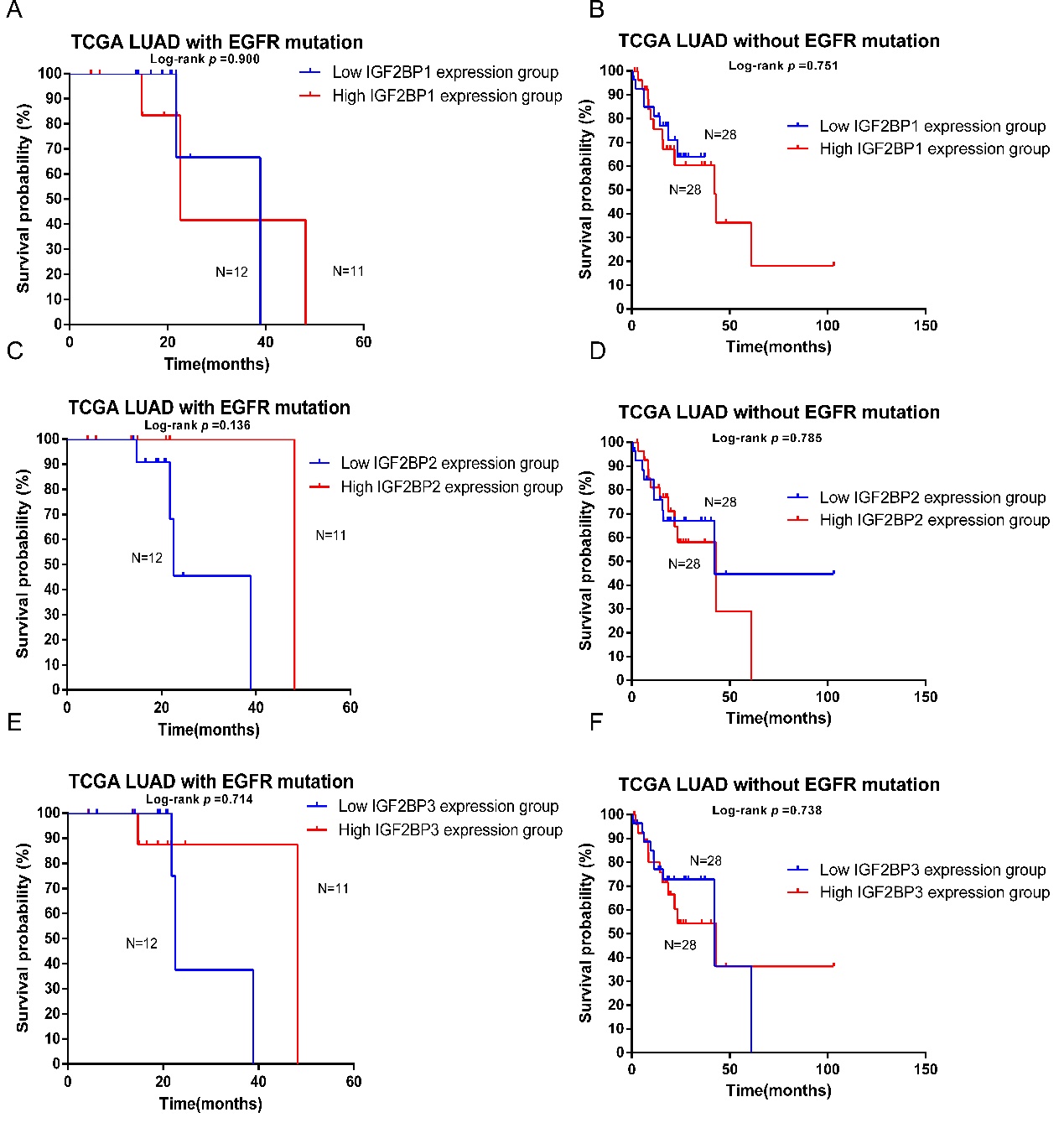
**

**Supplemental Figure 8**. Kaplan-Meier overall survival analysis of IGF2BPs in LUAD patients with or without EGFR mutations from the TARGET dataset.


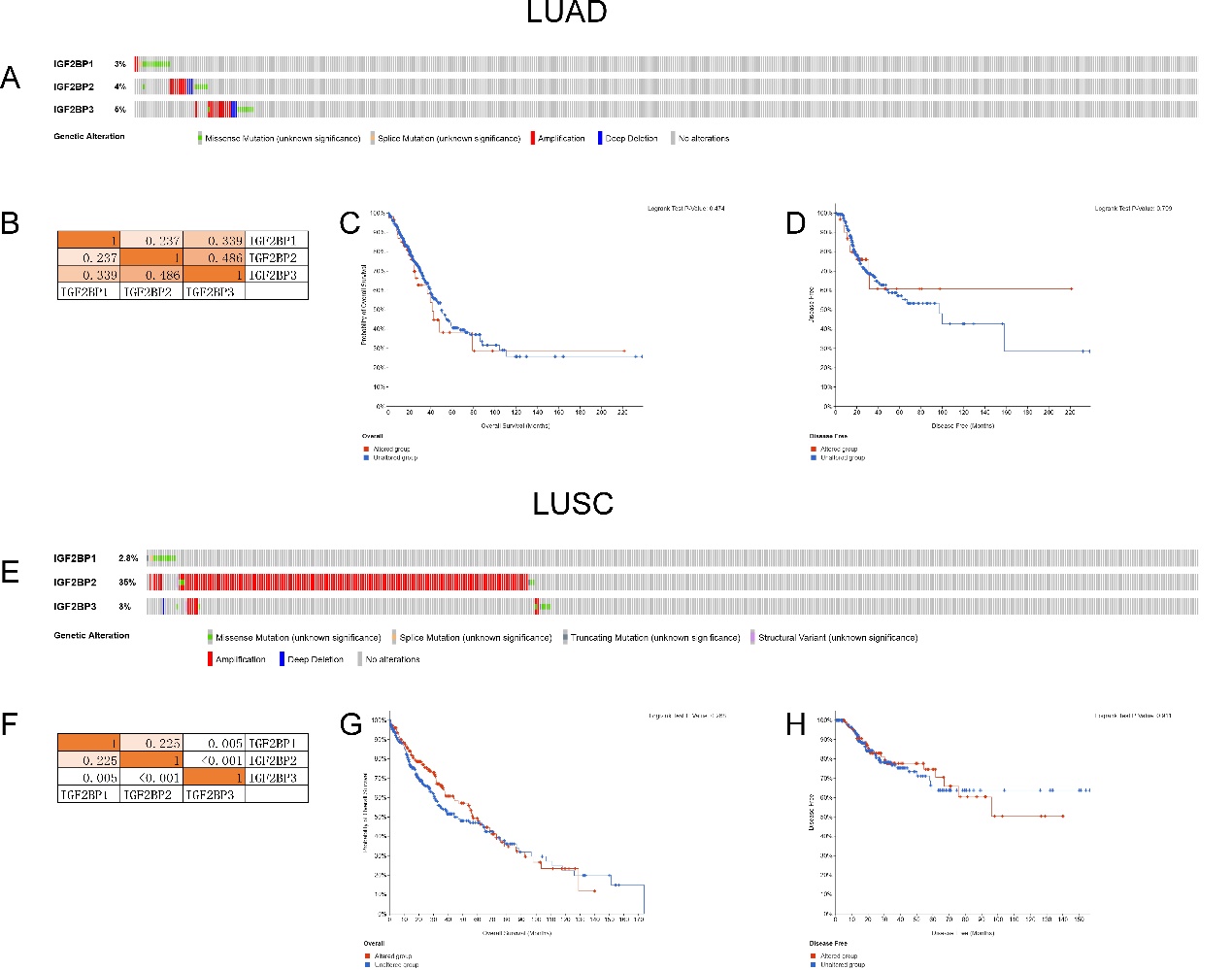


**Supplemental Figure 9.** Genetic mutations of IGF2BP family genes and their associations with OS and DFS in LUAD and LUSC (cBioPortal). (A) General genetic mutations of different IGF2BP family members in LUAD. (B) Correlations of distinct IGF2BP family members with each other in LUAD. OS and (C) DFS (D) curves of LUAD patients with or without genetic alterations of IGF2BP family. (E) General genetic mutations of different IGF2BP family members in LUSC. (F) Correlations of distinct IGF2BP family members with each other in LUSC. OS and (G) DFS (H) curves of LUSC patients with or without genetic alterations of IGF2BP family.

| **Supplemental Table 1.** Detailed information about those datasets in OSluca program | | | | | |
| --- | --- | --- | --- | --- | --- |
| **Datasets** | **Sample size** | **Tumor subtypes** | **Survival** | **Sources** | **Platform** |
| GSE10245 | 58 | LUAD/LUSC | OS | GEO | GPL570 |
| GSE12428 | 34 | LUSC | OS | GEO | GPL1708 |
| GSE13213 | 117 | LUAD | OS | GEO | GPL6480 |
| GSE14814 | 133 | LUAD/LUSC/NOS | OS | GEO | GPL96 |
| GSE17710 | 56 | LUSC | OS | GEO | GPL9053 |
| GSE19188 | 82 | LUAD/LUSC/LCC | OS | GEO | GPL570 |
| GSE26939 | 116 | LUAD | OS | GEO | GPL9053 |
| GSE29013 | 55 | LUAD/LUSC | OS | GEO | GPL570 |
| GSE29066 | 68 | LUAD/LUSC/SCLC | OS | GEO | GPL6947 |
| GSE30219 | 293 | LUAD/LUSC/SCLC/LCC | OS | GEO | GPL570 |
| GSE31210 | 226 | LUAD | OS | GEO | GPL570 |
| GSE3141 | 111 | LUAD/LUSC | OS | GEO | GPL570 |
| GSE31908 | 30 | LUAD | OS | GEO | GPL96 |
| GSE37745 | 196 | LUAD/LUSC/LCC | PFI | GEO | GPL570 |
| GSE41271 | 275 | LUAD/LUSC/LCC | OS | GEO | GPL6884 |
| GSE4573 | 139 | LUSC | OS | GEO | GPL96 |
| GSE50081 | 181 | LUAD/LUSC/LCC | OS | GEO | GPL570 |
| GSE5123 | 51 | LUSC | OS | GEO | GPL3877 |
| GSE5843 | 48 | LUAD | OS | GEO | GPL3877 |
| GSE67639 | 1106 | LUAD/LUSC/NOS | OS | GEO | GPL570 |
| GSE68465 | 442 | LUAD | OS | GEO | GPL96 |
| GSE68571 | 86 | LUAD | OS | GEO | GPL80 |
| TCGA | 1011 | LUAD/LUSC | OS | TCGA | DCC |
| Abbreviations: LUAD, lung adenocarcinoma;LUSC, lung squamous cell carcinoma;SCLC, small cell lung cancer; LCC, large cell cancer; NOS, not other specified; OS, overall survival; PFI, progression=free interval. | | | | | |
